# Breastfeeding Interruption and Resumption: A Risk-Based Clinical Decision Framework

**DOI:** 10.3390/jcm15208148

**Published:** 2026-10-20

**Authors:** Antoneta Dacia Petroaie, Alexandru Carauleanu, Iustina Petra Solomon Condriuc, Eric Oliviu Cosovanu, Elena Teona Cosovanu, Oana Raluca Temneanu, Ana Maria Slanina, Elena Popa, Adorata Elena Coman

**Affiliations:** 1 Grigore T. Popa University of Medicine and Pharmacy, 700115 Iasi, Romania; antoneta.petroaie@umfiasi.ro (A.D.P.); condriuc.iustina-petra@d.umfiasi.ro (I.P.S.C.); cosovanu_eric-oliviu@d.umfiasi.ro (E.O.C.); cosovanu_elena-teona@d.umfiasi.ro (E.T.C.); temneanu.oana@umfiasi.ro (O.R.T.); ana.slanina@umfiasi.ro (A.M.S.); elena.popa@umfiasi.ro (E.P.); elena.coman@umfiasi.ro (A.E.C.); 2 Department of Obstetrics and Gynecology, Cuza Voda Hospital, 700038 Iasi, Romania

**Keywords:** breast feeding, lactation, milk, human, contraindications, contrast media, radiopharmaceuticals, infectious disease transmission, vertical, clinical decision-making

## Abstract

Lactating women often need medication, contrast-enhanced or nuclear imaging, or treatment for infection, and each raises the question of whether breastfeeding can continue. Advice is frequently binary, and in some settings interruption may be recommended more readily than the evidence warrants. This narrative review proposes that breastfeeding interruption be managed as a clinical decision with three linked components: whether interruption is necessary, its minimum necessary duration and the criterion for resumption. We distinguish the hazard of an agent, the infant’s actual exposure and the resulting clinical risk, and apply a common sequence of questions to maternal pharmacotherapy, contrast media, radiopharmaceuticals and selected maternal infections, using pharmacokinetic, dosimetric or transmission measures. The evidence base remains limited and heterogeneous: in a major lactation database, 54% of listed medications had no breastfeeding safety data, and no direct measurement of contrast-media absorption in breastfed infants was identified. The proposed framework grades management across five levels, from unrestricted breastfeeding to avoidance or definitive cessation; whether a restriction is temporary or definitive follows the applicable guidance, and resumption criteria are defined at the outset. This conceptual aid is not a validated decision rule, does not replace agent-, procedure-, radionuclide- or pathogen-specific guidance and requires prospective evaluation.

## 1. Introduction

Breastfeeding is the reference standard for infant feeding. The American Academy of Pediatrics recommends exclusive breastfeeding for approximately the first six months, with continued breastfeeding alongside complementary foods thereafter [1]. The World Health Organization recommends exclusive breastfeeding for the first six months of life, followed by nutritionally adequate and safe complementary foods with continued breastfeeding up to two years of age or beyond [2]. Meta-analytic evidence links longer breastfeeding to lower infectious morbidity, reduced malocclusion and higher measured intelligence in the child. Probable reductions in overweight and diabetes are reported alongside an increase in dental caries [3]. For the mother, breastfeeding protects against breast cancer, improves birth spacing and may also protect against ovarian cancer and type 2 diabetes [3]. These benefits are graded, not binary, so shortening breastfeeding duration carries an incremental loss of protection [3]. Coverage remains far from the recommendations. In low- and middle-income countries, only 37% of infants under six months were exclusively breastfed. Duration is, with few exceptions, shorter in high-income countries than in resource-poor ones [3]. Practice is shaped by structural and commercial determinants that lie outside the mother-infant dyad [4,5].

Clinical events that raise doubt about continued breastfeeding are common during lactation. A systematic review of studies conducted mainly in high-income countries found that the proportion of postpartum women, breastfeeding or not, who used at least one medicine ranged from 34% to 100%; most studies reported more than 50% [6]. Diagnostic imaging, including contrast-enhanced studies and nuclear medicine procedures, may become necessary at any point during lactation [7], and maternal infection may also arise during this period. These situations require clinicians to determine whether breastfeeding can continue safely.

Advice is frequently binary: unrestricted continuation or complete cessation, with little defined ground between them. In a prospective Greek cohort of 847 women, 57 discontinued breastfeeding because of medication. Only 10.5% of these had received evidence-based counseling, and 68.4% had stopped on professional advice that the investigators considered erroneous [8]. A 2025 systematic review similarly suggested that much medication-related discontinuation may be avoidable, because most implicated drugs are compatible with lactation [9]. Women may likewise be advised to discard milk after imaging procedures that do not require interruption [7,10].

This pattern may reflect the conflation of three quantities that risk assessment keeps separate. A hazard is the intrinsic capacity of an agent to cause harm, whereas infant exposure is the fraction of that agent which actually reaches the infant through milk or contact. Clinical risk is a third quantity: the probability that such exposure produces a detectable adverse outcome. For medicines, exposure is commonly expressed as the milk-to-plasma ratio or the relative infant dose (Section 4.2), although neither is a stand-alone safety threshold [11]. A drug may be pharmacologically potent yet transfer poorly into milk and then be destroyed in the infant gut. The hazard is real, but the resulting clinical risk may be negligible. Recommendations based predominantly on intrinsic hazard may therefore overestimate the need for interruption.

The converse also holds: a small quantified exposure does not by itself imply negligible clinical risk. Idiosyncratic toxicity, hypersensitivity, very high pharmacological potency, cumulative or irreversible harm, active metabolites and severely impaired infant clearance can each break that inference (Section 4.3 and Section 4.4).

We propose that interrupting breastfeeding be regarded as a clinical intervention with its own physiology and potential harms, including downregulation of milk synthesis (Section 3.1), engorgement, ductal narrowing, breast refusal and difficulty resuming direct breastfeeding [7,10]. A clinical intervention normally has an indication, a defined duration and an explicit criterion for stopping, but interruption is seldom specified in these terms, and this omission may contribute to avoidable breastfeeding discontinuation.

Previous work has quantified medication-related discontinuation [9] and documented variation in practice after imaging [10]. At the point of care, clinicians rely on resources specific to an agent, a procedure or a disease. For medicines, these are lactation databases such as LactMed and the product information, supplemented where available by professional or national guidance; for imaging and infection, they are radiology protocols and public health guidance [7,12,13]. Milk4baby, a decision tool with a different aim, guides the choice of methods for studying infant exposure to medications through human milk [14]. Some resources already group recommendations across domains into broad categories [13], and others grade the compatibility of individual agents or conditions (Section 10.2); such resources primarily classify situations. This narrative review aims to make explicit the reasoning that links, across drugs, imaging-related exposures and maternal infections, the indication for interruption, its minimum necessary duration and the criterion for resumption. It applies a common sequence of questions, from exposure pathway to potential harm, while recognizing that each domain answers them with its own measures. From these questions it derives a proposed five-level management framework in which interruption is one option among several. The aim is proportionate decision-making: avoiding unnecessary interruption while supporting restriction where it is indicated.

The scope of the review and its exclusions are defined in Section 2.1; the review complements rather than replaces the agent-, procedure-, radionuclide- or pathogen-specific resources used at the point of care.

## 2. Materials and Methods

### 2.1. Review Design and Scope

We conducted a narrative review using a structured, purposive approach to literature identification and synthesis. Its objective was to bring three bodies of evidence that differ in kind into a single clinical decision structure. These are pharmacokinetic data on the transfer of drugs and contrast media into human milk, radiation dosimetry for radiopharmaceuticals, and transmission studies for maternal infection. Because they share no common effect measure, the synthesis was conceptual and integrative in purpose. The manuscript was prepared and critically appraised with reference to the six items of the Scale for the Assessment of Narrative Review Articles (SANRA) [15]. Accordingly, the search is described, the criteria for source selection are stated and the type of evidence behind each conclusion is identified. The review is narrative rather than systematic and does not claim exhaustive identification of the published literature.

The review covers three domains in which temporary interruption of breastfeeding is routinely considered: maternal pharmacotherapy; iodinated and gadolinium-based contrast media together with radiopharmaceuticals; and selected maternal infections. Infant-related conditions that preclude breastfeeding irrespective of maternal exposure were excluded, because they concern initiation rather than interruption. Maternal exposures that may require avoidance or definitive cessation were included as the boundary condition of the graded framework set out in Section 8.

### 2.2. Literature Search

We searched PubMed/MEDLINE, Scopus, Web of Science Core Collection, Embase and the Cochrane Library from database inception to 3 August 2026. No lower date limit was applied, because the pharmacokinetic and dosimetric literature underlying several currently quoted assumptions extends to the 1980s. Terms for breastfeeding, lactation and human milk were combined with terms for interruption, cessation and resumption, and searches were run separately for each domain. Two authors independently conducted the PubMed literature searches. The sources retained for the narrative synthesis and the key evidence used in the manuscript were subsequently cross-checked by two additional authors. This process strengthened source identification and verification; source selection remained relevance-based and purposive rather than a duplicate systematic screening against prespecified eligibility criteria.

Titles and abstracts of retrieved records were reviewed for relevance to the determinants examined in Section 4, and potentially relevant full texts were then examined. Reference lists of retrieved reviews, protocols and guidance documents were hand-searched for primary sources the databases did not return, particularly for the older pharmacokinetic and dosimetric studies underlying frequently quoted figures. Sources identified in either way were assessed against the clinical domains defined in Section 2.1 and the inclusion and exclusion criteria set out below; relevance was judged by the authors rather than against prespecified eligibility criteria. Language was restricted to English, French and Romanian.

The objective was to identify and map the evidence relevant to the framework; selection was therefore purposive and integrative rather than exhaustive. Sources were included when they informed at least one of the following: breastfeeding interruption, continuation or resumption; the pathway by which a maternal exposure reaches the infant; the magnitude and duration of infant exposure; infant susceptibility; the severity and reversibility of potential harm; strategies that reduce exposure without interrupting breastfeeding; or guidance relevant to interruption and resumption. Studies from adjacent fields, including mammary physiology, developmental pharmacology, radiation dosimetry and clinical decision-making, were included where they informed one of these elements. The evidence priorities applied are described in Section 2.4. Older pharmacokinetic and dosimetric studies were retained where they constituted the primary evidence underlying a quantitative assumption still cited in current guidance, as for the milk-transfer studies of contrast media examined in Section 6. The infections examined in Section 7 were selected as decisional archetypes representing distinct transmission pathways and management principles. Sources were excluded when they concerned exposures outside the three domains or infant conditions that preclude breastfeeding irrespective of maternal exposure, and secondary summaries were not used as the source of a recommendation when the primary guidance was available (Section 2.3). These criteria followed from the design of the review rather than from a prespecified protocol. The database-specific search strings and the numbers of records retrieved are not available and are therefore not reported; because selection was purposive and iterative rather than a screening of a fixed set of records against prespecified eligibility criteria, numbers of included studies comparable to those of a systematic review do not apply, and the reference list comprises the sources cited in support of the synthesis rather than a systematically included set of studies.

### 2.3. Selection of Guidelines and Authoritative Lactation Resources

Recommendations were extracted from primary guidance rather than from secondary summaries. For pharmacotherapy we consulted LactMed [12] alongside the relevant European summaries of product characteristics. For contrast media and radiopharmaceuticals we used the Academy of Breastfeeding Medicine protocol on radiology and nuclear medicine [7] together with International Commission on Radiological Protection dosimetry. For infection we used current national and supranational public health guidance, principally that of the Centers for Disease Control and Prevention (CDC), the U.S. Department of Health and Human Services (HHS), the World Health Organization (WHO) and the Pan American Health Organization. Authoritative clinical guidelines were obtained from the publications and websites of the issuing organizations, separately from the database search, and the most current versions available during manuscript preparation were used. Where two authoritative sources disagreed, the discordance was recorded rather than resolved, because such disagreement is itself an object of this review. During revision, the statements on therapeutic radioiodine and on infection-specific guidance were rechecked against the current versions of the cited guidance [7,12,13], and five sources were added outside the original database search: three classification resources used to position the framework against existing tools (Section 10.2), an experimental study of active transport into milk (Section 5.2) and a 2026 guideline on thyroid disease in preconception, pregnancy and the postpartum period, cited for its recommendations on radionuclide procedures and radioiodine therapy during lactation (Section 6.4).

### 2.4. Approach to Evidence Synthesis

Evidence was prioritized according to its suitability for the specific question rather than by a single design hierarchy. Systematic reviews and comparative clinical studies were prioritized for clinical outcomes, pharmacokinetic studies for milk transfer and infant exposure, dosimetric studies for radionuclide exposure, transmission studies for maternal infection, and current authoritative guidance for clinical management recommendations. A randomized trial is not inherently more informative than a pharmacokinetic study when the question is transfer into milk, or than dosimetry when the question is radionuclide exposure.

Given the heterogeneity of evidence types and the conceptual rather than guideline-development purpose of this review, a formal certainty-of-evidence grading system was not applied. Formal grading presupposes a systematically identified body of evidence and outcomes that can be compared across studies; neither condition applies to a review that brings together milk-transfer pharmacokinetics, radiation dosimetry and transmission studies, each addressing a different question. Instead, the type and the limitations of the evidence supporting clinically relevant conclusions are stated explicitly at the point each conclusion is drawn. Where a conclusion rests on modeling, dosimetry or consensus rather than on clinical observation, the text says so. The evidential basis is described with the following categories: direct clinical or infant-outcome evidence, human pharmacokinetic or milk-transfer measurement, radiation dosimetry, observational transmission data, authoritative clinical guidance, physiological reasoning, and indirect or extrapolated evidence. These categories describe the nature of the evidence; they are not ranked and do not express certainty. Where relevant, we indicate whether a value or criterion is measured, calculated, assumed, taken from guidance, established by clinical verification, or left to clinical judgment. Section 10.1 summarizes the evidential basis and principal limitation of the major propositions of the review and distinguishes recommendations reproduced from authoritative guidance from propositions synthesized by the authors. Synthesis proceeded qualitatively and by domain.

The framework presented in Section 8 was developed iteratively from the determinants set out in Section 4 and from the guidance reviewed in Section 5, Section 6 and Section 7, and its consistency with that guidance was checked in representative situations from each domain (Section 8.6). This process is not clinical validation: the framework has not undergone prospective, external, outcome-based or formal consensus validation, and it does not supersede current agent-, procedure-, radionuclide- or pathogen-specific guidance. Section 10.3 outlines how the framework could be evaluated.

## 3. Breastfeeding Interruption as a Clinical Intervention

Advising a mother to interrupt breastfeeding is an active clinical decision that carries its own consequences for lactation and for the feeding relationship.

### 3.1. Why Interruption Is Not a Neutral Decision

The rate of milk synthesis is controlled locally, within each breast, by the frequency and completeness of milk removal. In direct measurements in lactating women, short-term synthesis rates varied markedly between the two breasts of the same mother and, in 6 of 13 breasts studied, were positively related to the degree of emptying [16]. Infants rarely emptied the breast, removing on average 76% of available milk [16]. Longer intervals yielded proportionally less milk: production was lower after gaps of 6 to 18 h than after gaps under 6 h (*p* < 0.0002) [17].

The mechanism is autocrine inhibition by a milk constituent. A whey protein isolated from caprine milk, the feedback inhibitor of lactation, reversibly suppresses synthesis in the epithelial cells that secrete it [18]. It has been characterized mainly in animal models, but the human studies above reproduce the same functional relationship: milk retained in the alveoli suppresses further production. Interrupting feeding without equivalent expression therefore signals the gland to reduce production.

Reduced milk removal can cause alveolar distention and congestion, and milk stasis has been postulated to contribute to the inflammation of the mastitis spectrum [19]. Substitute feeding may also compromise return to the breast: even short periods of bottle use have been linked to breastfeeding problems and discontinuation, and short interruptions to breast refusal [10]. In an online cross-sectional survey of 392 women, 34.9% had interrupted breastfeeding, and 39.4% of these relactated successfully [20]. Because the data are self-reported, with recall of up to 36 months, this proportion is indicative only. It nonetheless suggests that resumption cannot be assumed. The expected consequences of interruptions of different duration are summarized in Table 1.

### 3.2. Decision-Making Under Uncertainty

Decisions about breastfeeding are made under uncertainty, and the consequences of the options are not equally visible: an adverse effect after continued breastfeeding is readily attributed to the decision, whereas reduced supply or early weaning after interruption may be delayed and less readily attributed to the advice.

In general decision research, harm from omission is often judged less blameworthy than equivalent harm from action [22], and in a randomized survey experiment, pulmonologists more often chose a suboptimal strategy when an option preserving the status quo was available [23]. Whether such tendencies influence lactation decisions has not been tested. We propose, as a hypothesis, that interruption may be perceived as the more conservative option despite its own physiological consequences (Section 3.1), a possibility we term the precautionary paradox. Asking whether a decision would be judged differently if the options were reversed has been proposed as a general corrective [24].

Limited knowledge may compound this tendency. Among 404 professionals surveyed about breastfeeding after radiological studies, 63% judged their own knowledge insufficient, and interruption was recommended more often after contrast-enhanced examinations (*p* < 0.01) [10]. The harm of unnecessary interruption has not, however, been quantified against the risk it is intended to avoid.

### 3.3. The Mother-Infant Dyad as the Unit of Clinical Decision-Making

A defensible decision weighs four quantities together: the risk of infant exposure, the necessity of maternal treatment, the consequences of interrupting lactation, and alternatives that reduce exposure without stopping. Optimizing any one in isolation can compromise the others.

A common difficulty is an exclusive focus on reducing infant exposure, which may leave maternal disease untreated. The consequences are not confined to the mother. A systematic review of 122 studies of untreated postpartum depression documented effects on infant sleep, growth, and motor, cognitive, language, emotional and behavioral development, as well as on bonding and breastfeeding [25]. Selective serotonin reuptake inhibitors were the second most frequently dispensed drug class in a cohort of lactating women [26]. Where mental health conditions arise during lactation, effective treatment of the mother should take priority [27]. Avoiding milk-borne exposure by leaving depression untreated exchanges one infant risk for another.

Protection conferred by human milk is graded by duration [3]. Because medicine use after delivery is common (Section 1), a rule that resolved every uncertainty by stopping could affect many breastfeeding dyads. Mother and infant are therefore assessed as one unit, and the decision is a trade-off between competing risks.

### 3.4. Temporary Interruption Versus Definitive Cessation

Temporary interruption and definitive cessation are distinct interventions, and this review uses the two terms strictly. The distinction may be lost in the advice given. Temporary interruption presupposes reversibility, which cannot be taken for granted.

Reversibility depends on the length of the gap, the infant’s age, maintained milk removal and the availability of skilled support. In a systematic review of 16 relactation studies, more than 80% of mothers resumed breastfeeding in 13 [21]. The interventions were intensive and structured, and the enabling factors were younger infant age, a shorter lactation gap, maternal motivation, family support and continuous skilled support [21]. The interventions mainly concerned hospitalized infants or healthy infants who were not breastfed, and reasons for stopping were rarely reported, so the figures apply to maternal medication, imaging or infection only by inference.

The survey and the relactation studies are not comparable intervention groups. They differ in population, reason for stopping, design and intensity of support, and no effect size should be derived from the contrast. Read together, they are nonetheless consistent with the view that outcome depends substantially on what accompanies an interruption [20,21]. A temporary interruption advised without a stated duration, maintained milk removal or a plan for resumption may function as unintended weaning.

## 4. From Maternal Exposure to Infant Clinical Risk

**Figure 1:**
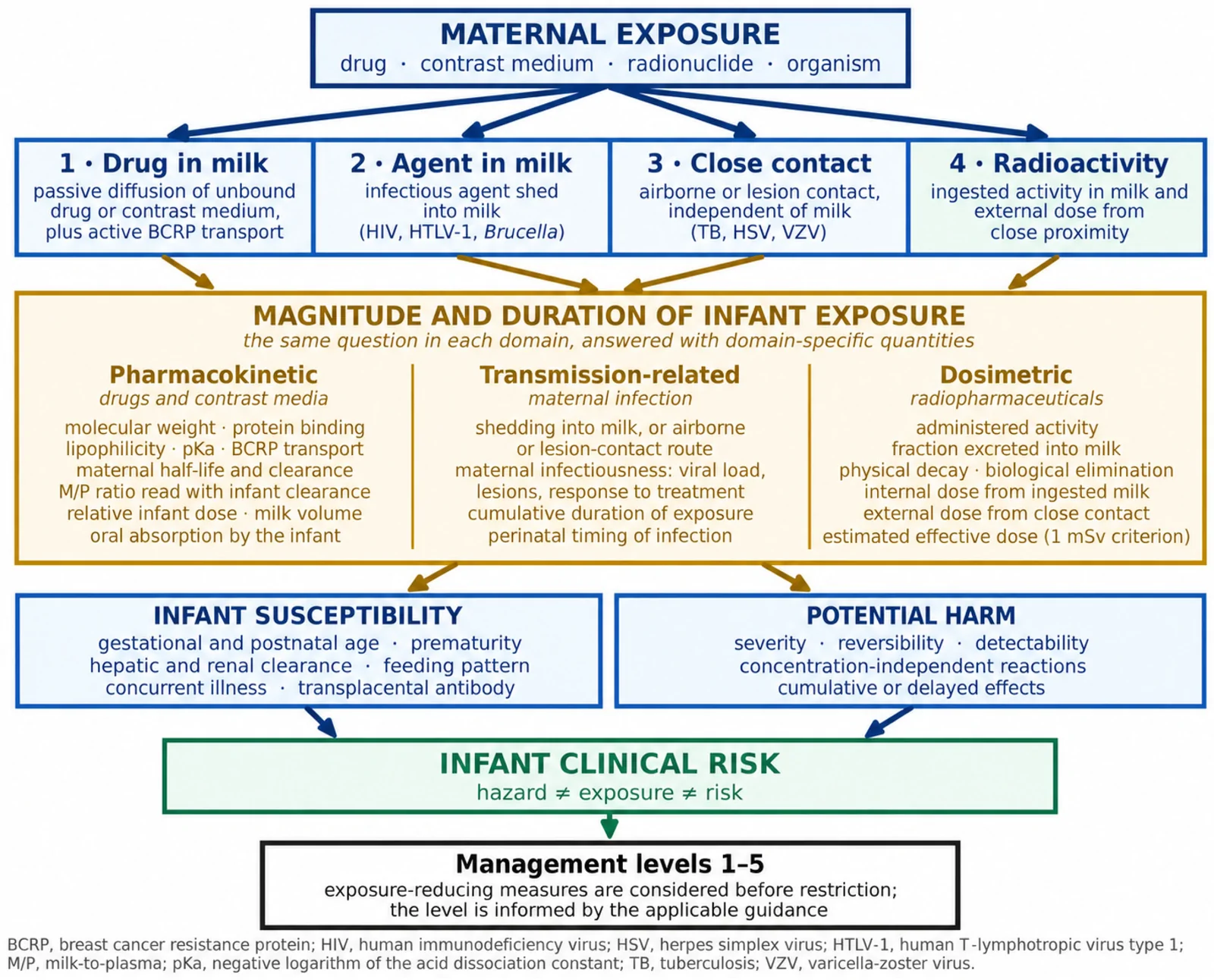
From maternal exposure to infant clinical risk: four exposure pathways; the domain-specific quantities used to estimate the magnitude and duration of infant exposure (pharmacokinetic for drugs and contrast media, transmission-related for maternal infection, dosimetric for radiopharmaceuticals); and the infant and harm-related determinants that convert exposure into clinical risk. The management level (Section 8) is also informed by the applicable guidance.

If interruption is an intervention, the first question is whether it is necessary. Answering it requires moving from what the mother received to what the infant actually faces. The determinants below distinguish hazard, infant exposure and clinical risk across the three domains. The questions they raise are common to all three, but the quantities used to answer them differ: pharmacokinetic for drugs and contrast media, dosimetric for radiopharmaceuticals, and transmission-related for maternal infection (Figure 1).

### 4.1. Identifying the Exposure Pathway

The assessment begins with the route by which the infant could be reached. Four pathways operate across the three domains (Figure 1). A drug may transfer from maternal plasma into milk. An infectious agent may be shed into milk. Transmission may occur through close maternal contact, independently of milk. Radioactivity travels by both routes at once, since a radionuclide excreted into milk is also a source of external exposure at close range.

Identifying the pathway determines which options remain. Where an agent is transmitted by contact rather than through milk, expressed milk may remain usable, subject to pathogen-specific guidance. Suspending feeding at the breast is not the same as withdrawing human milk, and this distinction can resolve many cases before any quantitative assessment. In maternal tuberculosis, for example, the concern is airborne transmission from an infectious mother, not the milk.

### 4.2. Estimating the Magnitude and Duration of Infant Exposure

The mammary gland does not act as a drug depot between feeds. The infant ingests what is present in milk at the time of feeding, so maternal plasma kinetics, together with the extent of transfer into milk, determine exposure [11,28].

The standard metric is the relative infant dose: the weight-adjusted daily intake through milk as a percentage of the weight-adjusted maternal dose [11]. A value below 10% is conventionally used as a reference point, on the reasoning that this fraction of a therapeutic dose is unlikely to produce dose-dependent effects. It is a convention for interpretation, not an outcome-derived threshold separating safe from unsafe agents [11].

Three misinterpretations are common. The first is treating the milk-to-plasma ratio as a safety index. The ratio is uninformative in isolation: infant exposure varies with the ratio divided by infant drug clearance, a hyperbolic dependence on clearance [29]. A high ratio for a rapidly cleared drug may therefore deliver only a small absolute quantity [11]. The second is equating milk concentration with systemic exposure. Oral bioavailability, gastrointestinal degradation, active metabolites and elimination half-life all intervene between milk and infant plasma. The third is treating detection as evidence of risk. Statements that a drug is excreted into the milk of lactating animals have been judged largely irrelevant to human lactation safety, because risk depends on quantity, not presence [28].

Ingested volume completes the estimate. Exclusively breastfed infants aged one to six months feed about 11 times daily, taking about 76 g and removing roughly two-thirds of the available milk at each feed [30]. For a similar daily intake, weight-adjusted exposure is greater in younger, lighter infants. In the first days, wide junctions between alveolar cells allow readier passage of drug into milk, but the small volume of colostrum limits the quantity ingested [27]. Duration of exposure depends on half-life or, for radionuclides, on physical decay and biological elimination.

### 4.3. Assessing Infant Susceptibility

Identical exposure does not carry identical risk. Drug disposition changes as metabolic capacity, sites of distribution and organ function mature [31]. Neonates, preterm infants and infants under two months of age have immature hepatic and renal function. Their reduced clearance permits accumulation from quantities that an older infant would eliminate without consequence. Where the function of eliminating organs is pathologically low, the relative infant dose cannot be applied at face value [11].

Feeding pattern modifies exposure independently of physiology: an exclusively breastfed infant ingests substantially more milk per kilogram than one receiving mixed feeding. Concurrent illness, prematurity and coadministered medication further alter susceptibility.

### 4.4. Severity and Reversibility of Potential Harm

Probability of an event is not sufficient for a decision; severity and reversibility should be weighed alongside it. A transient, observable and reversible potential effect, such as mild sedation or altered stool pattern, may permit continued breastfeeding with defined monitoring, depending on the agent, exposure magnitude and infant characteristics. Sedation warrants particular caution in neonates, preterm infants and infants with impaired clearance, in whom accumulation is more likely (Section 4.3). An effect that would be irreversible by the time it became apparent does not permit this approach.

Detectability is therefore a decision variable in its own right. Where an adverse effect would be recognized early and would resolve on withdrawal, continuation with defined monitoring may be defensible even under uncertainty. Two exceptions limit this reasoning. Some reactions are unrelated to concentration, allergic and hypersensitivity responses among them, so reducing exposure does not abolish them [11]. Others become clinically apparent only after cumulative exposure, which shifts the relevant assessment from single-dose to duration.

### 4.5. Identifying Exposure-Mitigation Strategies

In the proposed framework, interruption is considered only after measures that reduce infant exposure while lactation continues. These measures derive from pharmacokinetic reasoning rather than comparative trials, a limitation shared with much of the guidance examined here.

Therapeutic substitution is usually considered first: for many drug classes, an alternative with lower milk transfer, a shorter half-life or poorer oral bioavailability can be selected [27], which may resolve the question without affecting the feeding relationship.

Where substitution is impossible, dose scheduling may help for selected agents. For short-half-life agents without clinically important active metabolites or accumulation, dosing immediately after a feed or before the infant’s longest anticipated sleep may reduce peak milk exposure [27]. The strategy offers little benefit for long-half-life or sustained-release agents, drugs with long-lived active metabolites or drugs that accumulate. For these, substitution or monitoring is the operative measure.

Route of administration offers a further margin: topical and inhaled preparations generally produce lower maternal plasma concentrations than systemic routes [27]. Preparations applied to the nipple are the exception, because they reach the infant directly [27].

Three further measures apply where exposure cannot be reduced at source. Milk expressed and stored beforehand can bridge a defined interval. Feeding from the unaffected breast is possible when a lesion is localized. Where a bank is accessible, donor human milk preserves human milk feeding when maternal milk must be withheld.

Where measurement is unavailable, prediction is increasingly viable. Milk pH and fat content limit how well physicochemical models estimate the milk-to-plasma ratio, and these limits are now characterized [32]. Population pharmacokinetic modeling of milk concentrations measured in lactating patients who were not breastfeeding allows extrapolation to realistic breastfeeding conditions [33].

Where none of these measures is applicable or sufficient, and defined monitoring cannot adequately manage the residual risk, restriction may be considered: temporary interruption where the applicable guidance supports it, with its minimum necessary duration and the criterion for resumption defined at the outset (Section 9), or avoidance or cessation where that guidance does not support resumption (Section 8.5).

## 5. Maternal Pharmacotherapy

Pharmacotherapy is a frequent context for these decisions and a domain in which the gap between evidence and advice has been studied directly [8,9].

### 5.1. Magnitude and Drivers of Medication-Related Interruption

Medicine use after delivery is common: in most studies included in a systematic review, more than half of postpartum women used at least one medicine [6]. In the United States, clinicians are described as frequently lacking the evidence needed to counsel a breastfeeding woman on a drug’s effects on her infant, on herself or on lactation [34]. Medication is therefore a frequent trigger for the question addressed here. Because so many women are affected, the quality of this advice matters for breastfeeding at the population level as well as for the individual dyad.

Medication is also a recognized reason for ending breastfeeding. In the Greek cohort described in Section 1, 39 of the 57 medication-related discontinuations followed professional advice that was not evidence-based [8]. A systematic review likewise suggested that much medication-related discontinuation may be avoidable [9]. These findings suggest that counseling quality is an important and potentially modifiable contributor to discontinuation.

### 5.2. Physicochemical Determinants of Transfer into Milk

Transfer into milk occurs mainly by passive diffusion of unbound drug down a concentration gradient, modified by the physicochemical properties of the molecule [28,35]. Four properties matter most. Molecular weight limits passage, so large molecules, including most monoclonal antibodies, reach milk in negligible quantities. Protein binding determines how much drug is free to diffuse, and highly bound agents transfer little. Lipophilicity governs partition into the lipid phase of milk. The dissociation constant matters because milk is slightly more acidic than plasma, so weak bases become ionized and accumulate in milk [28].

Partitioning theory alone predicts only the unbound ratio and estimates whole-milk concentrations poorly, because drug also distributes into the protein and lipid phases of milk [36]. A model predicting milk-to-plasma ratios from these physicochemical characteristics was later evaluated prospectively in 10 basic drugs [37]. Prediction, however, is not measurement. Measurement in lactating women, even in relatively few subjects, provides valuable quantification of transfer [28], and a predicted milk concentration is not a predicted infant exposure (Section 4.2).

Passive diffusion does not account for every agent. The breast cancer resistance protein is strongly induced in the mammary gland during lactation and, in mice, actively secretes substrates such as topotecan and cimetidine into milk [38]. In lactating women, milk concentrations of cimetidine and nitrofurantoin exceed those predicted from passive diffusion [35]. Models based on molecular properties alone will misestimate transfer for such substrates.

Molecular weight operates most visibly at the opposite extreme. In a review covering 19 monoclonal antibodies, 30 studies sampling 155 women reported typically low milk concentrations, the highest milk-to-serum ratio being 1:20, for infliximab [39]. For certolizumab pegol the relative infant dose fell between 0.04% and 0.30% [40]. Clinical outcome data point the same way. Among infants of women with inflammatory bowel disease, breastfed and non-breastfed infants had similar infection rates (39% in each group) and developmental milestone scores (87 and 86), and outcomes did not differ significantly between infants exposed and those not exposed to biologic, immunomodulator or combination therapy [41].

A further limitation, distinct from the misinterpretations described in Section 4.2, concerns the metric itself. Relative infant dose is popular because it requires only milk sampling, avoiding venepuncture in mother and infant, and because published cut-off values make interpretation simple [42]. For monoclonal antibodies, however, values are frequently miscalculated, and the metric may not suit the drug class [43]. Proper estimation requires sampling across the dosing interval to approximate the area under the milk concentration-time curve, which few published values do [42].

### 5.3. Discordance Between Product Labeling and Lactation Resources

Direct human evidence remains limited for many lactation recommendations. Of 1408 medications listed in LactMed in 2018, 54% had no breastfeeding-related safety data and only 2% had sound safety evidence [34].

**Figure 2:**
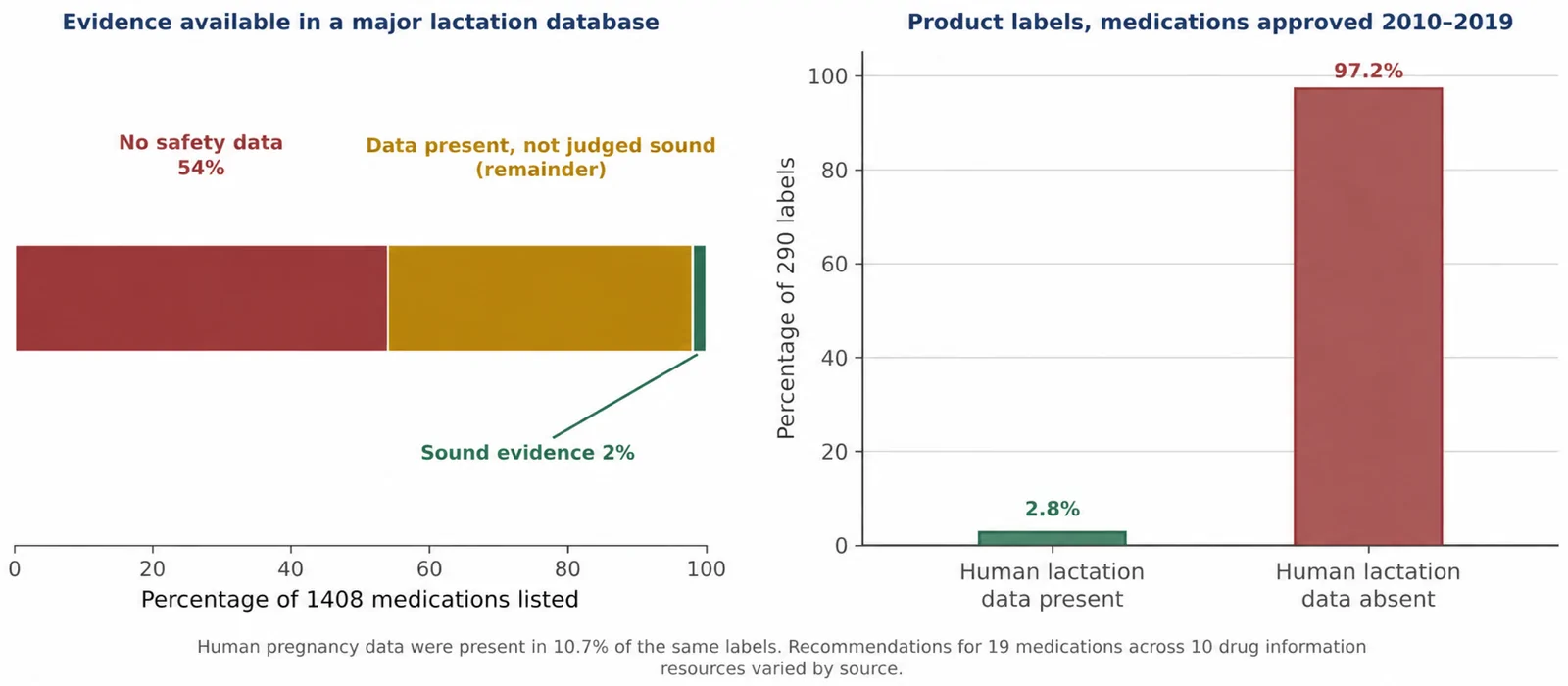
Evidence base underlying lactation guidance. (**Left**) Distribution of evidence quality across 1408 medications listed in a major lactation database; the intermediate segment is the remainder after the two reported categories and is not itself a reported value. (**Right**) Presence of human lactation data in the labels of 290 medications approved between 2010 and 2019; such data were present in 2.8% of labels. Resources may interpret limited human lactation evidence differently.

Resources may therefore reach different conclusions from the same evidence. When recommendations for 19 medications were compared across 10 drug information resources, the degree of compatibility assigned varied by source, and dedicated lactation references classified the most agents as compatible [44]. A clinician consulting two resources may obtain two answers. The divergence may reflect different evidentiary thresholds, update cycles or regulatory and clinical purposes. It does not follow that all positions are equally well supported. Table 2 compares the evidential basis of each source type, and Figure 2 illustrates the underlying evidence limitations.

Product labeling is often the most conservative of these sources and the one the prescriber encounters first. Labels and lactation-specific databases serve different purposes, and a label may favor conservative wording when direct human lactation data are unavailable. As noted in Section 4.2, statements about excretion into animal milk carry little weight for human lactation safety [28].

Of 290 medications approved between 2010 and 2019, human lactation data appeared in 2.8% of labels and human pregnancy data in 10.7%. Of 138 products submitted before the Pregnancy and Lactation Labeling Rule took effect in 2015, 45 (32.6%) were still not in the required format at the June 2019 deadline [45].

Where a label and an evidence-based lactation resource diverge, the divergence may reflect these differing purposes and evidentiary thresholds rather than a new safety signal. Treating it as a safety signal may lead to unnecessary interruption.

### 5.4. Clinical Patterns Organized by Decision

Pharmacological class alone does not determine management, so the categories below are defined by clinical action. They correspond to the management levels developed in Section 8.

The first three categories permit continued breastfeeding: without qualification, with defined monitoring, or after exposure reduction by substitution, dose timing or a change of route.

The fourth category comprises temporary interruption of defined duration, supported by the applicable guidance, with milk removal maintained and a stated criterion for resumption. The fifth comprises avoidance or definitive cessation where that guidance does not support resumption.

**Figure 3:**
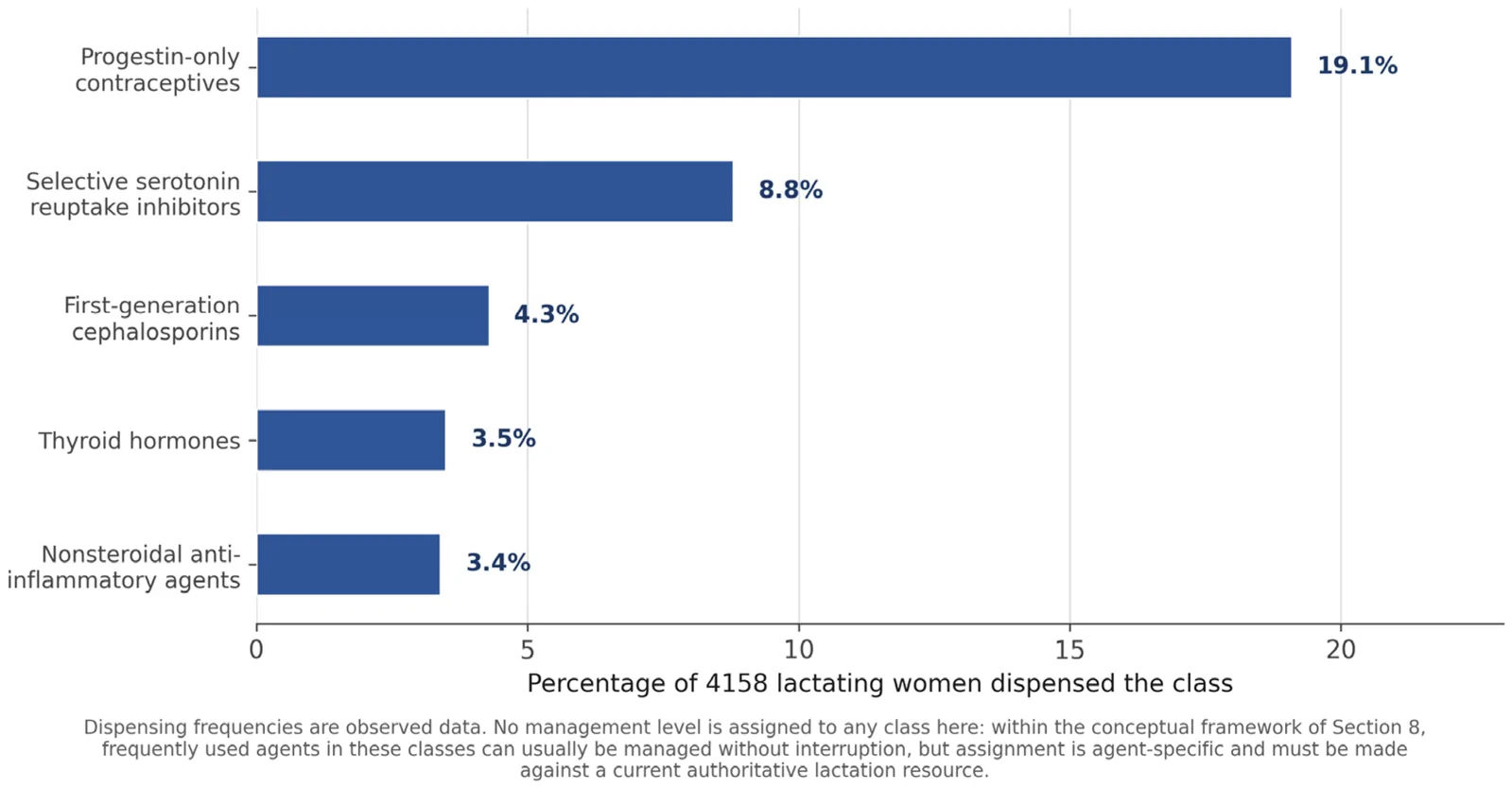
The five classes most frequently dispensed to 4158 lactating women [26]. The figure reports dispensing frequency only; no management level is assigned to any class, because the framework proposed in Section 8 operates on individual agents, which require assessment against a current authoritative lactation resource.

These categories apply to the drugs that lactating women actually receive. In a cohort of 4158 lactating women, the five classes most frequently dispensed were progestin-only contraceptives (19.1%), selective serotonin reuptake inhibitors (8.8%), first-generation cephalosporins (4.3%), thyroid hormones (3.5%) and nonsteroidal anti-inflammatory agents (3.4%) [26]. For most frequently used agents in these classes, management is generally possible without complete interruption (Figure 3). No management level can be assigned to a class as a whole, however, and the level for an individual agent should be determined against a current authoritative lactation resource. Table 3 defines the five levels by clinical action.

No management level can be assigned to a class as a whole, however, and the level for an individual agent should be determined against a current authoritative lactation resource. Table 3 defines the five levels by clinical action.

### 5.5. Agents Requiring Interruption or Definitive Cessation

This category is small, and its membership changes as data accumulate. Determinations should therefore be made against a current primary resource at the time of prescribing [12], not from a fixed list.

Pharmacovigilance data indicate where scrutiny is warranted. Among 2628 lactation-related adverse event reports submitted to the United States Food and Drug Administration between 2001 and 2024, 50.9% of the reported drugs acted on the nervous system and 19.9% were antineoplastic and immunomodulating agents [46]. Because the denominator is unknown and reporting is voluntary, these proportions cannot establish incidence or show that either class is uniformly incompatible with breastfeeding. The frequency of adverse reactions in breastfed infants has been characterized as lower than commonly assumed [47].

Three patterns of reasoning underlie the guidance that places an agent in this category. The first is cytotoxicity. Most sources regard cytotoxic antineoplastic therapy as incompatible with concurrent breastfeeding [12], although the supporting evidence is thin and few studies have assessed it directly [48]. Agent-specific reasoning has begun to qualify the categorical rule. Population pharmacokinetic modeling now allows estimation of milk concentrations for individual cytotoxic agents [33]. For cyclophosphamide, the calculated interval to a cumulative relative infant dose below 1% is one to two days after each dose [12]. Such modeling does not establish a clinically validated resumption interval where toxic metabolites or infant outcomes are not adequately characterized. For cyclophosphamide, the potentially toxic metabolites were not measured [12].

The second pattern is a narrow therapeutic index combined with immature infant clearance, so that a quantity tolerated by an older infant may accumulate to effect in a neonate. The third is an elimination profile so prolonged that no feasible measure brings infant intake below concern. Radiopharmaceuticals, governed by physical decay, biological elimination and radiation dosimetry, are treated separately in Section 6.

### 5.6. Neonates, Preterm Infants and Reduced Clearance

Reduced clearance in early infancy permits accumulation from quantities an older infant would eliminate without consequence (Section 4.3).

Pharmacovigilance data are consistent with this physiology: 68.4% of the lactation-related adverse event reports described above concerned infants under two months of age [46]. Such reports cannot establish incidence, and the limitations of this database have been set out explicitly [49]. They do indicate that recognized events cluster steeply in early infancy.

The same agent may therefore warrant monitoring in a two-week-old and none in a four-month-old. A recommendation made in the neonatal period should be revisited as the infant matures.

## 6. Contrast Media and Radiopharmaceuticals

Two classes of agent are often grouped under imaging, but they follow different logic. Contrast media are chemical substances, so the reasoning of Section 5 applies: what matters is how much transfers into milk and how much of that is absorbed. Radiopharmaceuticals are radioactive sources, for which the governing quantity is absorbed dose rather than molar concentration. Plain radiography, computed tomography without contrast, ultrasonography and magnetic resonance imaging without gadolinium deliver nothing to the infant through milk and require no breastfeeding precautions [7].

### 6.1. Iodinated Contrast Media

Infant exposure to iodinated contrast passes two sequential barriers. Less than 1% of the maternal dose is excreted into milk within 24 h, and less than 1% of the quantity ingested is assumed to be absorbed from the infant gut. The estimated systemic dose to the infant is therefore below 0.01% of the maternal intravascular dose, which is under 1% of the dose that infant would receive if imaged directly [50]. On this basis breastfeeding may continue without interruption, and expressing and discarding milk is not required [50].

The conclusion is sound, but its evidential foundation is narrower than its frequent restatement implies. The first figure derives from small studies of iohexol, metrizoate and metrizamide conducted in the 1980s [51,52], from which a nursing infant was calculated to receive 0.5% of the maternal dose [53]. The second, the assumption that under 1% of ingested contrast is absorbed, is less well supported. No direct measurement of gastrointestinal absorption of contrast media in breastfed infants was identified, and a PubMed keyword search for such data located eight publications, all of them reviews or guidelines mentioning the figure only in their discussion [53]. The estimate is therefore an extrapolation from outside the breastfed-infant setting, and the relative immaturity of the infant intestinal barrier has been raised as a reason why the absorbed proportion might differ [54]. Not every iodinated agent has been measured in human milk, although guideline wording can give that impression [54]. Reported iodine concentrations in milk span zero to 36.5 mg/L over 24 h [55], and the published measurements of iodinated contrast in human milk derive from 22 lactating women, most of whom received agents no longer marketed [53].

This does not overturn the recommendation, because the available measurements do not materially alter the underlying risk assessment [50,56]. Acknowledging the uncertainty matters for another reason. A clinician who finds that the package insert contradicts the guideline may be more persuaded by a recommendation that explains the discrepancy than by one that does not acknowledge it [54]. In one survey, 68% of respondents recommended discarding milk for 24 to 48 h after one or both agent types, and 95% requested formal guidance [57].

### 6.2. Gadolinium-Based Contrast Agents

**Figure 4:**
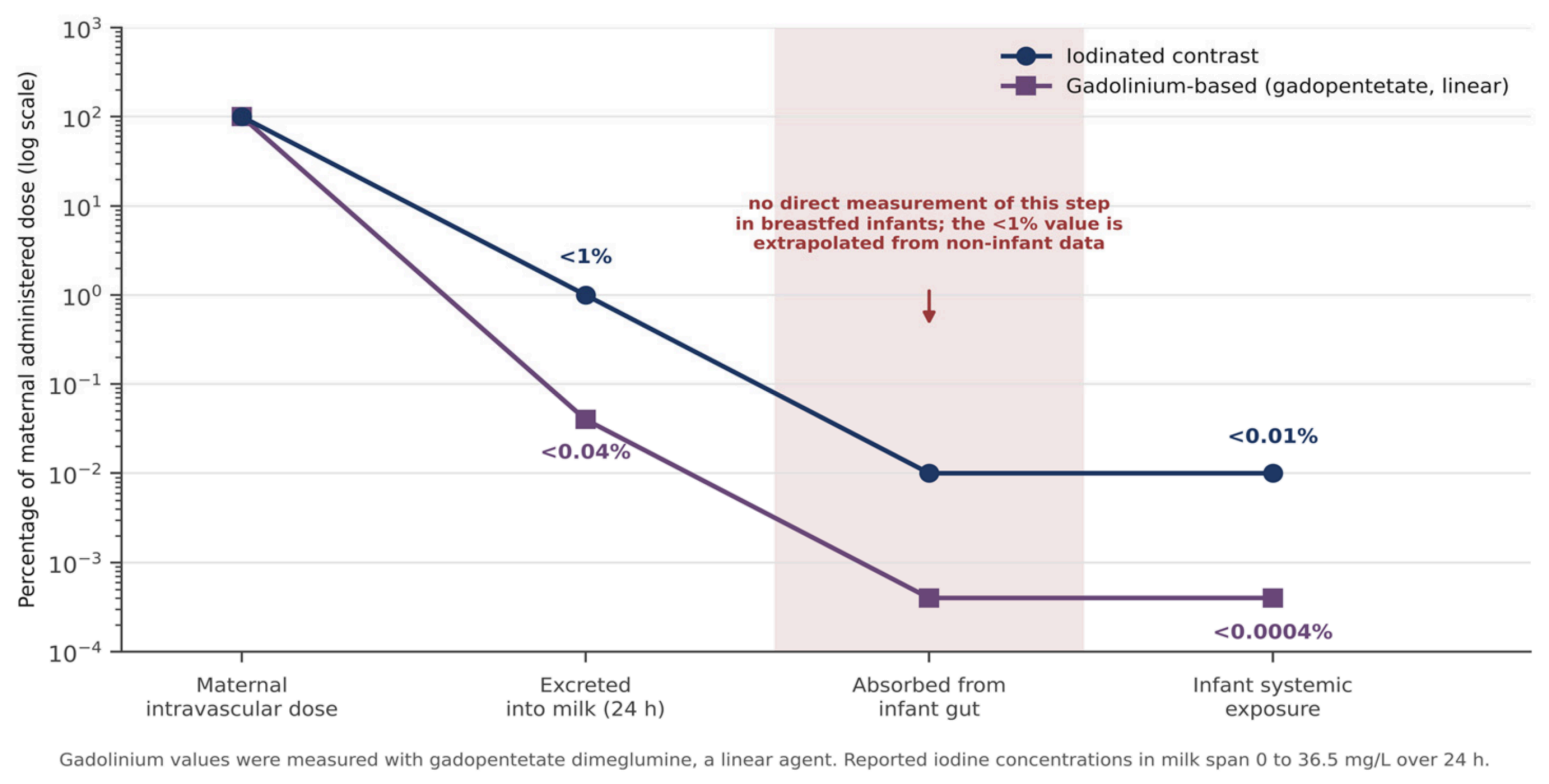
Sequential attenuation of infant exposure to iodinated and gadolinium-based contrast media, expressed as a percentage of the maternal intravascular dose on a logarithmic scale. The shaded step marks the absorption assumption, for which no direct measurement in breastfed infants was identified and which is an extrapolation from outside the breastfed-infant setting. Gadolinium values derive from measurements with gadopentetate dimeglumine, a linear agent; human milk measurements for macrocyclic agents were not identified [54].

Gadolinium-based agents transfer less. In human studies of gadopentetate dimeglumine, a linear ionic agent, under 0.04% of the maternal intravascular dose appeared in milk within 24 h [58], and under 1% of that quantity is assumed to be absorbed. Estimated systemic exposure is therefore below 0.0004% of the maternal dose [50]. Figure 4 shows the attenuation at each step for both agent classes.

These measurements come from one series of 20 women [58] and two single-patient reports [59,60], all with gadopentetate dimeglumine, and no human milk measurements for macrocyclic agents were identified [54]. European guidance states that breastfeeding may continue normally after macrocyclic gadolinium-based agents [56], although the milk-transfer data on which such recommendations draw were obtained with a linear agent. A joint position paper of Italian professional bodies considered breastfeeding compatible with the administration of most contrast media [61]. The same evidential caveat applies as for iodinated agents, since the absorption assumption is shared [54].

### 6.3. Radiopharmaceuticals and Nuclear Medicine Procedures

For a radiopharmaceutical, risk depends on the administered activity, the biokinetics of the compound and the resulting radiation dosimetry rather than on mass concentration in milk. Two exposure routes operate concurrently. Activity excreted into milk is ingested and delivers an internal dose, whereas activity retained in maternal tissues delivers an external dose during close contact. Interruption of feeding addresses only the first route. The second is addressed, where material, by limiting close contact for a specified interval (Section 6.4).

The International Commission on Radiological Protection published biokinetic and dosimetric models for 33 radiopharmaceuticals, with recommendations on breastfeeding interruption [62], and later updated its dose compendium [63]. Its interruption intervals are calibrated to keep the estimated effective dose to the infant below 1 mSv, a radiation-protection criterion that the Academy of Breastfeeding Medicine also uses as a general rule [7]. The dose compared with this criterion is estimated from dosimetric models rather than measured, and the criterion is not a boundary between safe and harmful exposure. In one of the largest published biokinetic datasets, dosimetric estimates indicated that most of the diagnostic radiopharmaceuticals studied required no interruption to meet this criterion [64].

**Figure 5:**
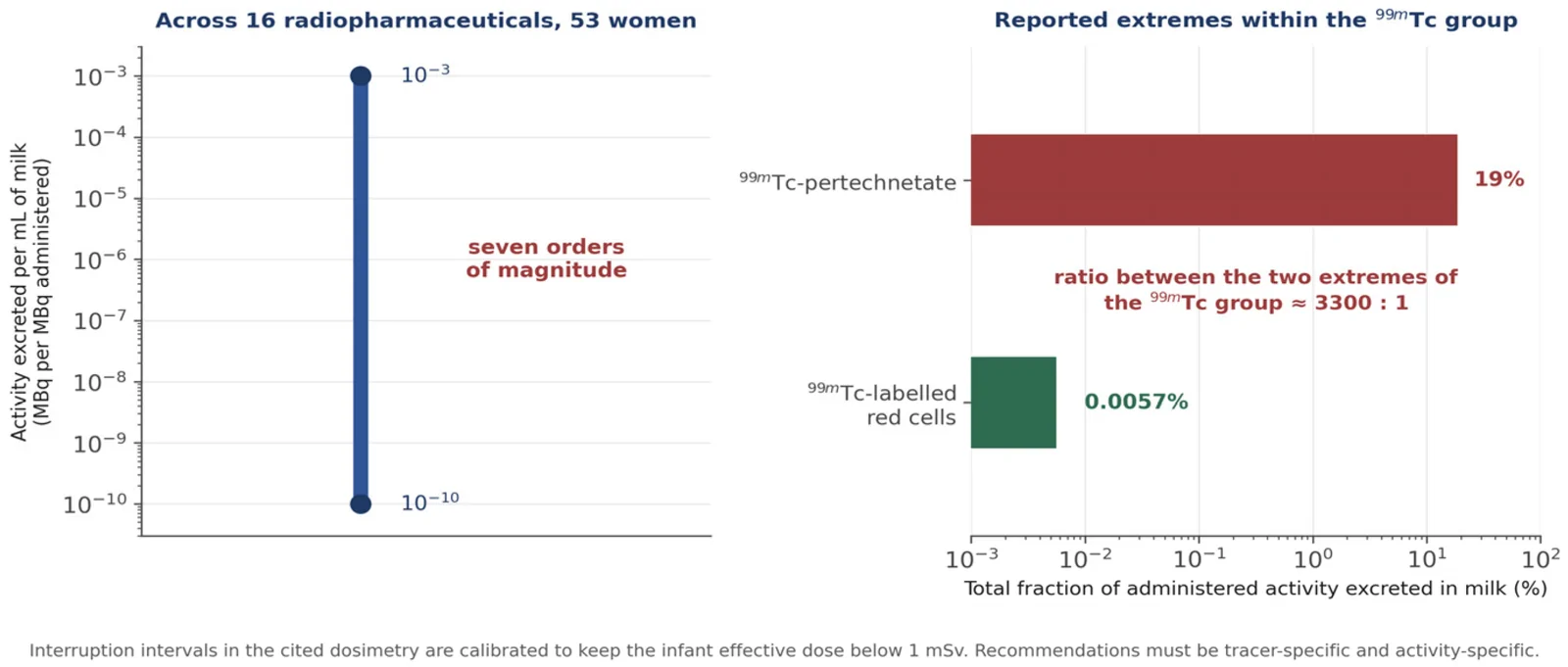
Radiopharmaceuticals do not behave as a class. Left: activity excreted per milliliter of milk across 16 agents sampled from 53 women, spanning seven orders of magnitude. Right: the reported extremes of the total excreted fraction within the technetium-99m group [64].

Radiopharmaceuticals do not behave as a class. In milk sampled from 53 women after 16 different agents, the activity excreted per milliliter spanned seven orders of magnitude, from 10^−10^ to 10^−3^ MBq per MBq administered [64]. The dispersion persists within a single radionuclide. Among technetium-99m compounds, the fraction of administered activity excreted in milk ranged from 0.0057% for labeled red blood cells to 19% for pertechnetate [64]. The corresponding estimated effective dose to the infant ranged from 6.7 × 10^−6^ to 3.6 × 10^−2^ mSv per MBq administered to the mother [64]. Across the other agents, the excreted fraction ranged from 0.018% for chromium-51 ethylenediaminetetraacetic acid (EDTA) to 48% for iodine-131 sodium iodide, with estimated effective doses from 5.6 × 10^−5^ to 106 mSv per MBq [64]. Cessation of breastfeeding was recommended for iodine-131 sodium iodide, which had the highest value [64]. Figure 5 displays this dispersion.

With differences of this magnitude, no instruction keyed to the procedure can suit all agents. A fixed interruption after any scan may be excessive for many tracers and insufficient for a few. An independent dosimetric review of 25 radiopharmaceuticals judged interruption necessary for only 9 [65]. It noted considerable variation in milk concentrations and effective half-lives between individuals [65].

### 6.4. Radionuclide-Specific Interruption Intervals

The required interval depends on four quantities: physical half-life, biological elimination, the fraction of administered activity excreted into milk, and the administered activity. Because these vary independently, the interval must be derived agent by agent from published dosimetry rather than inferred from the imaging modality [62,63,66]. Where retained maternal activity contributes materially to the infant dose, a separate restriction on close contact may apply, not necessarily for the same period [65,66].

These datasets distinguish three groups of agents. On dosimetric estimates, many diagnostic tracers required no interruption against the 1 mSv criterion. A smaller group required interruption measured in hours to days. In the biokinetic series described above, 12 h of interruption was recommended for iodine-125 iodohippurate, iodine-131 iodohippurate, technetium-99m pertechnetate and technetium-99m macroaggregated albumin, with milk expressed at least three times and discarded [64]. For technetium-99m pertechnetate, Academy of Breastfeeding Medicine guidance makes the interval depend on the administered activity: 12 to 24 h, and as little as 4 h after 185 MBq [7]. For diagnostic thyroid scintigraphy in the evaluation of hyperthyroidism during lactation, the 2026 guideline of the American Thyroid Association recommends that milk be expressed and discarded for 36 h after technetium-99m pertechnetate and for 3 to 4 days after iodine-123 [67]. For pertechnetate, these sources give intervals of different kinds: one derived from measured excretion and dosimetric estimates in a single biokinetic series [64], an activity-dependent protocol recommendation [7] and a procedure-specific guideline recommendation [67]. They are not interchangeable, and none defines a universal biological threshold. A third group warrants cessation, and the note to Table 4 distinguishes the two iodine radionuclides. Agent-specific intervals are tabulated in the dosimetric publications and are not reproduced exhaustively here. Recommendations must be tracer-specific and activity-specific, because technetium-99m compounds do not behave as a single group. Recent society guidance agrees. The 2026 stress-testing guideline of the American Society of Nuclear Cardiology advises interrupting breastfeeding and close physical proximity for 24 h after technetium-99m sestamibi or tetrofosmin, with the breasts emptied once and the milk discarded [68]. For fluorine-18 flurpiridaz, it notes that the prescribing information specifies at least 8 h, whereas the multi-society procedure standard specifies at least 12 h [68]. Recommendations may therefore differ between authorities, reflecting differences in assumed administered activity, dose criteria and clinical scope rather than necessarily conflicting measurements.

### 6.5. The Pump-And-Dump Concept

Expressing and discarding milk serves two purposes that are often conflated: removing contaminated milk, and maintaining production and preventing stasis during a temporary interruption. Advice should state which one applies.

After contrast administration, neither purpose applies: the quantity of contrast in milk is not considered clinically relevant, and production is not at risk because feeding continues [50]. A mother who prefers to discard milk may do so, although routine discarding is not required [50].

After radiopharmaceutical administration the position differs by agent. Discarding removes ingestible activity, which is worthwhile where the excreted fraction is substantial, and pointless where it is negligible. Regular expression also removes activity from the breast and so contributes to the biological elimination of the radiopharmaceutical, and guidance for diagnostic thyroid scintigraphy specifies that milk be expressed and discarded during the interval [67]. Expression does not, however, alter physical decay, and it should not be used to shorten an established interval without an agent-specific assessment. The radiation dose to the lactating breast itself is a separate consideration from the infant’s exposure; it underlies the advice to stop breastfeeding weeks to months before therapeutic iodine-131 (Table 4).

Where temporary interruption is indicated, expression is needed to maintain lactation, the second purpose. Milk should be removed at about the infant’s usual feeding frequency to limit the autocrine downregulation and stasis described in Section 3.1, whether or not it is discarded. Advice to interrupt without instruction to express is one way in which a temporary interruption may become permanent.

## 7. Selected Maternal Infections

The infections below were selected because each illustrates a different decisional principle; together they show that the route of transmission resolves many management questions.

Where transmission occurs through milk, the milk is the object of the decision; where it occurs by respiratory or contact routes, proximity is usually the main issue, although pathogen-specific guidance may still restrict expressed milk [13]. Table 5 summarizes the infections examined and the principle each illustrates.

### 7.1. HIV: Risk Modified by Treatment and Viral Suppression

Perinatal guidance on human immunodeficiency virus (HIV) infection in high-income settings has changed within the last three years, reflecting evidence that transmission risk depends on treatment rather than being a fixed property of infection. Previously, replacement feeding was the only recommended option in the United States; national guidance has since replaced it with patient-centered counseling and shared decision-making, because sustained viral suppression on antiretroviral therapy (ART) reduces transmission through breastfeeding to below 1%, though not to zero [69]. At the time of writing, counseling on formula feeding, banked donor milk or breastfeeding is offered when ART has been taken consistently and the viral load has been below 50 copies/mL for at least three months before delivery [69]. In the largest randomized comparison, transmission on maternal ART was 0.3% at six months and remained below 1% at twelve months of breastfeeding [75].

Two qualifications apply. In the maternal ART arm of that trial, viral load at delivery or in the first postpartum week was below the assay limit of detection in 41% of women and detectable but below 400 copies/mL in a further 23%, and most received a lopinavir-based regimen [75]. More consequentially, the principle that an undetectable viral load renders a person untransmittable does not extend to breastfeeding: transmission has been documented despite sustained maternal virological suppression [76], and in the randomized trial, two infants in the maternal ART arm acquired HIV although maternal viral load was undetectable or below 40 copies/mL [69].

Guidance therefore attaches conditions. Replacement feeding remains recommended where ART is not being taken or suppression has not been achieved during pregnancy and at delivery, and CDC guidance advises against breastfeeding when sustained viral suppression cannot be maintained after birth [13]. If the viral load becomes detectable during breastfeeding, the HHS panel advises stopping temporarily or discontinuing while the cause is assessed, and most experts advise permanent discontinuation at or above 200 copies/mL [69]. In Europe, most of 19 guidelines from 20 countries name formula as the preferred option while supporting parents who choose to breastfeed under defined criteria [77], and roughly half of responding countries reported more women with HIV choosing to breastfeed [78].

**Figure 6:**
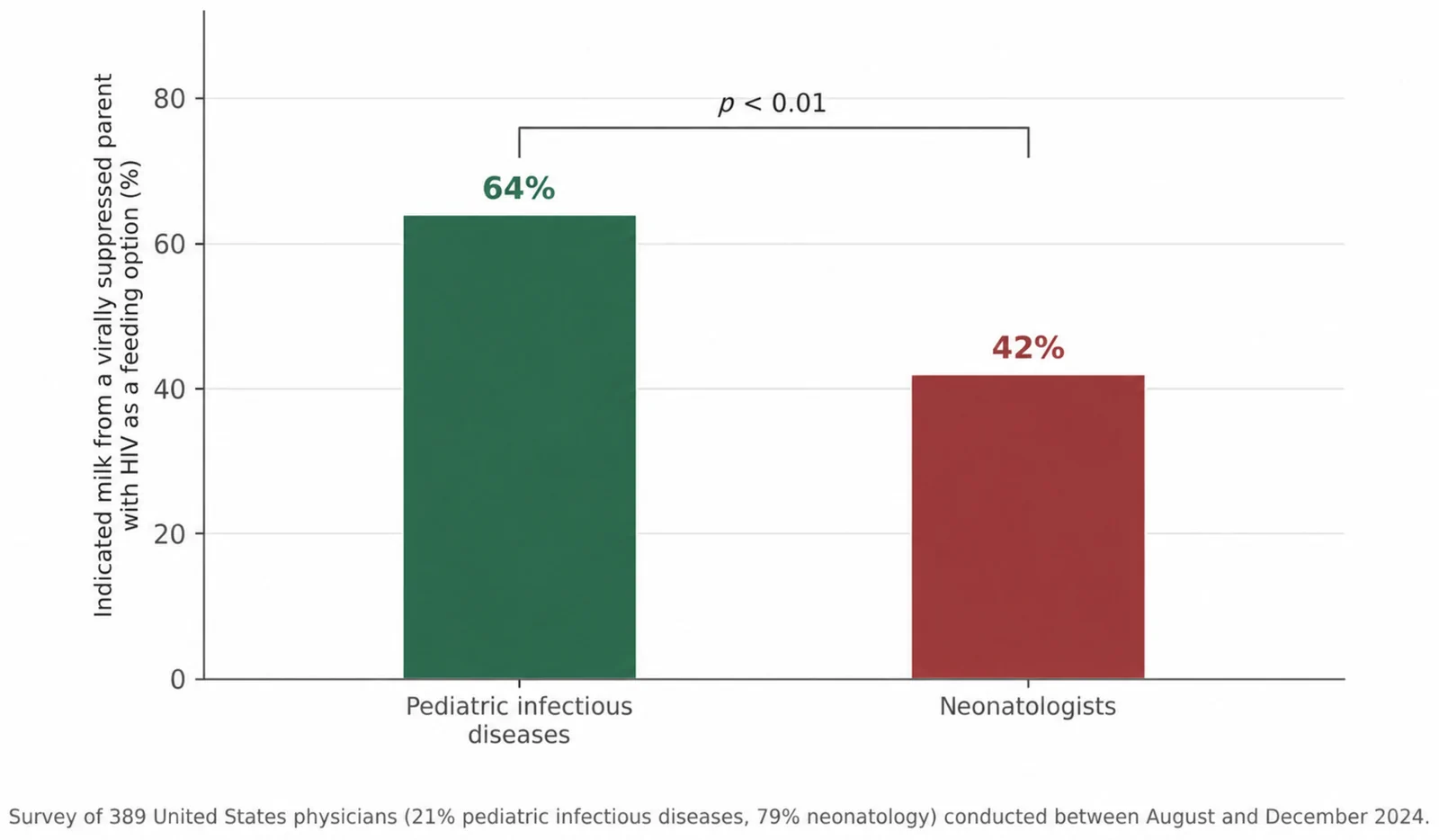
Proportion of surveyed physicians indicating milk from a virally suppressed parent with HIV as a feeding option, by specialty, among 389 United States respondents surveyed between August and December 2024 [79].

In a 2024 survey of 389 United States neonatologists and pediatric infectious disease physicians, 64% of infectious disease specialists and 42% of neonatologists indicated milk from a virally suppressed parent with HIV as a feeding option (*p* < 0.01) [79], so changes in guidance may not be uniformly reflected in practice (Figure 6).

### 7.2. HTLV-1: Cumulative Milk-Mediated Exposure

Human T-lymphotropic virus type 1 (HTLV-1) illustrates a different pattern. Roughly 95% of mother-to-child transmission is attributable to prolonged breastfeeding, so the exposure variable is duration rather than occurrence [80].

**Figure 7:**
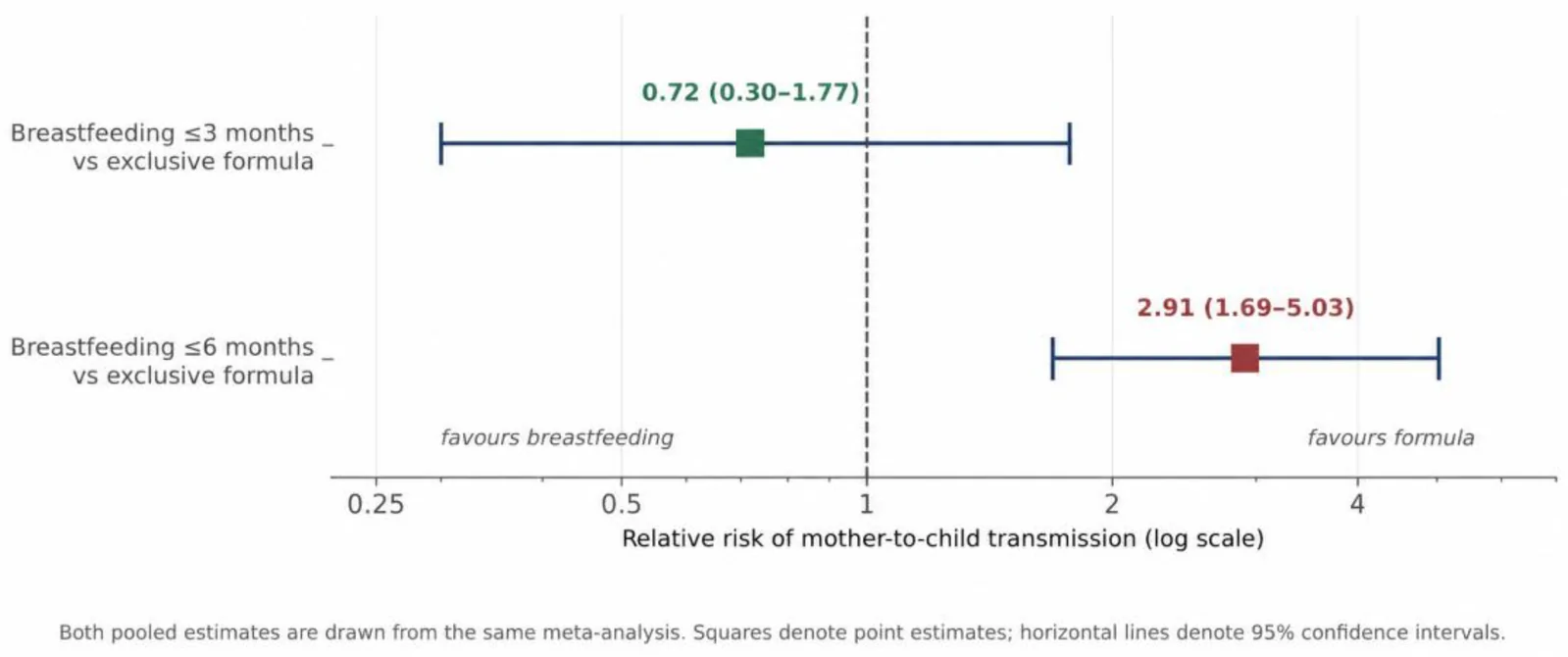
Pooled relative risk of HTLV-1 mother-to-child transmission for breastfeeding of three months or less and of six months or less, each relative to exclusive formula feeding, from the same meta-analysis [70]. Squares denote point estimates; horizontal lines denote 95% confidence intervals.

In a prospective multicenter Japanese cohort, transmission occurred in 4 of 172 infants (2.3%) breastfed for three months or less and in 7 of 110 (6.4%) fed exclusively with formula, a non-significant difference [70]. In the meta-analysis that incorporated this cohort, the pooled relative risk versus exclusive formula feeding was 0.72 for breastfeeding of three months or less (95% confidence interval, 0.30–1.77) and 2.91 for six months or less (1.69–5.03) [70]. Figure 7 displays both estimates. In a separate synthesis not stratified by duration, transmission occurred in 17.5% of breastfed and 4.3% of exclusively formula-fed infants, and avoiding breastfeeding was estimated to prevent about 85% of transmissions [81].

The same cohort exposes an operational problem: among mothers who chose short-term breastfeeding, 33.5% were still breastfeeding at three months and 7.8% at six months [82]. An intention to stop at a defined point is not the same as stopping, so a time-limited plan may need explicit follow-up [81]. A WHO technical report identifies breastfeeding as the main route of mother-to-child transmission [83], but authoritative guidance on infant feeding diverges. Japanese national guidance names exclusive formula feeding as the first option [82]; CDC lists HTLV-1 or human T-lymphotropic virus type 2 (HTLV-2) infection among the conditions in which breastfeeding and expressed maternal milk are to be avoided [13]; and a Pan American Health Organization technical note confines short-term breastfeeding, ideally for up to three months, to settings where formula feeding is not acceptable, feasible, affordable, sustainable and safe [84]. Management is therefore guideline-dependent.

HIV and HTLV-1 also intersect: women with HIV who are supported to breastfeed may carry unrecognized HTLV-1, which antenatal screening rarely covers, and systematic HTLV-1 screening has been proposed for women living with HIV, particularly those from endemic regions, to address this emerging risk [85].

### 7.3. Tuberculosis: Maternal Contagiousness Versus Milk Safety

Maternal tuberculosis illustrates that separation and withdrawal of human milk are distinct interventions. Untreated active pulmonary disease can reach the infant by the airborne route during close contact, whereas in the absence of breast involvement, breast milk is not considered a route of *Mycobacterium tuberculosis* transmission, and only small, non-toxic quantities of first-line antituberculous agents pass into it [71].

Direct feeding at the breast is therefore suspended while the mother remains infectious, but expressed milk may be given by another caregiver throughout; CDC guidance links resumption of direct breastfeeding to at least two weeks of appropriate treatment and physician documentation that the mother is no longer contagious [13]. The quantity of drug transferred is insufficient to treat or prevent tuberculosis in the infant, although the pharmacokinetic data are sparse [71,86].

### 7.4. Herpes Simplex: Anatomical Site Determines Management

Herpes simplex localizes the decision to an anatomical site: transmission during breastfeeding has been attributed to contact with lesions on the breast, which may go unrecognized [72].

CDC guidance lists herpes simplex as a reason to withhold breastfeeding and expressed milk only when lesions are on the breast [13], although active lesions elsewhere on the body should be covered. Feeding from the affected side and use of its expressed milk are then withheld until healing [13,72], whereas feeding from the unaffected breast may continue if lesions on the affected breast are completely covered [13]. Herpetic mastitis is itself most often acquired from an infected infant during feeding [87].

Where lesions are extensive, maintained expression can preserve lactation through a prolonged healing period (Section 9.6) [88].

### 7.5. Varicella: Timing-Dependent Neonatal Risk

Varicella has a narrow perinatal risk window with an immunological basis: maternal rash appearing between five days before and two days after delivery leaves too little time for transplacental transfer of protective antibody [89]. In the defining cohort of 281 newborns whose mothers had perinatal chickenpox, immunoglobulin G (IgG) antibody was present at birth in every infant born more than seven days after onset of the maternal rash and in none born within three days of onset [90].

Infection was common: of 280 surviving infants given varicella-zoster immunoglobulin, 169 (60%) became infected, 134 (48%) with clinical chickenpox, and the clinical attack rate was highest among infants whose mothers developed chickenpox between seven days before and seven days after delivery [90].

Within this window, CDC guidance advises temporarily suspending direct breastfeeding while allowing expressed milk to be given; outside it, the restriction does not apply [13]. The guidance does not state when direct breastfeeding may resume, which therefore rests on clinical judgment that the mother is no longer infectious; the window defines neonatal risk, not a calendar endpoint for resumption. Perinatal maternal herpes zoster did not cause neonatal infection in the same cohort [90], so the restriction is specific to primary varicella.

### 7.6. Brucellosis, Hepatitis and Emerging Infections

Brucellosis occupies a middle position. Infant infections possibly associated with breastfeeding have been reported, *Brucella* has been isolated from milk several times [91], and a 2025 family cluster describes an exclusively breastfed infant infected while consuming no other implicated product [92]. CDC guidance lists untreated maternal brucellosis among the indications for temporarily withholding both breastfeeding and expressed milk [13]. The restriction applies to untreated infection, but the guidance states neither an interval nor a criterion for resumption; initiation of treatment does not by itself establish that breastfeeding can resume, and the timing of resumption requires individualized specialist assessment (Section 8.4). Lactation can be preserved by expression during the restriction.

For Ebola virus disease, the recommendation to stop breastfeeding is supported by direct, although very limited, infant outcome data. In a systematic review that included eight studies of ten breastfeeding mothers, virus was detected in seven of ten milk samples, and four of the five infants fed virus-positive milk became infected; all four died [93]. Because virus is also recovered from other body fluids and contaminated surfaces, milk cannot be established with certainty as the principal route [93]. Virus was isolated from breast milk 15 days after symptom onset, and viral RNA remained detectable at 17 days [94]. WHO recommendations issued in February 2020 advise stopping breastfeeding where Ebola virus disease is suspected or confirmed in a lactating woman or her breastfeeding child [73,95]. Stopping is not necessarily permanent: after recovery and clearance of viremia, a woman who wishes to resume breastfeeding should wait until her milk contains no detectable Ebola virus RNA on two consecutive reverse transcription polymerase chain reaction tests 24 h apart, with a breast milk substitute in the interval [73,95]. Testing the milk itself matters, because a negative blood test does not necessarily mean that the milk is free of virus [96]. CDC guidance, by contrast, advises against breastfeeding and expressed milk in suspected or confirmed Ebola virus disease and states no criterion for resumption [13].

Mpox shows how authoritative guidance can diverge when direct evidence is limited. CDC guidance withholds breastfeeding and expressed milk until the criteria for ending isolation are met, including resolution of all lesions, detachment of scabs and a fresh layer of intact skin [13]. The 2025 WHO living guideline instead conditionally recommends, on low-certainty evidence, that mothers with mpox continue breastfeeding with infection-prevention measures while limiting direct contact with a non-infected infant, without feeding from a breast with areolar lesions [74]. The two sources share the endpoint for full resumption, complete resolution of lesions, but differ before it, and management follows the guidance in force in the setting.

Hepatitis B is managed by neonatal immunoprophylaxis and, where indicated, maternal antiviral therapy, rather than by feeding restriction [97,98,99]. The current practice guideline of the American Association for the Study of Liver Diseases and the Infectious Diseases Society of America states that breastfeeding does not increase the risk of hepatitis B virus transmission and considers tenofovir disoproxil fumarate or tenofovir alafenamide safe during lactation [99], and CDC guidance states that mothers with hepatitis B can breastfeed and reports no documented evidence of hepatitis C transmission through breastfeeding [98]. For both viruses, data are insufficient to establish whether feeding may continue when the nipple or surrounding areola is cracked and bleeding; because both are transmitted by blood, the recommendation is then to stop breastfeeding temporarily and to express and discard milk until healing is complete, after which feeding may fully resume, with expression maintaining supply in the interval [98].

## 8. A Proposed Risk-Based Framework for Breastfeeding Management

The three domains examined above differ in their physics and their evidence. They share a common sequence of questions (Section 4), but each domain answers them with its own evidence: pharmacokinetic and toxicological data for drugs and contrast media; biokinetics, dosimetry, physical decay and external exposure during close contact for radiopharmaceuticals; and, for maternal infection, the transmission pathway, infectivity, treatment status and pathogen-specific criteria for resumption. What is common is the reasoning process, not a quantitative scale.

The proposed structure is a continuum of five management levels, ordered by increasing restriction of the feeding relationship (Table 3). In the decision pathway (Section 8.6), modification and defined monitoring are both considered before restriction. The documentation suggested for the clinician increases as management becomes more restrictive.

The framework is a conceptual aid derived from the reviewed evidence and guidance. It has not been validated prospectively, externally or by formal consensus; it is not a clinical prediction model, and its levels are categories of clinical action rather than fixed numerical thresholds. It is intended to support rather than prescribe clinical decision-making; where it appears to diverge from current authoritative guidance for a particular agent, procedure, radionuclide or pathogen, the guidance governs.

### 8.1. Unrestricted Breastfeeding

Many common situations fall within this level. Breastfeeding proceeds without qualification where no plausible exposure route exists, or where quantified exposure is very low and no qualifying consideration from Section 4.4 applies.

Examples from the preceding sections include imaging without contrast, iodinated and gadolinium-based contrast agents [50] and most diagnostic radiopharmaceuticals on the dosimetric estimates of one of the largest biokinetic datasets [64], subject to tracer-specific guidance (Section 6.4). They also include hepatitis B and C unless the nipples are cracked and bleeding [98,99], herpes simplex without breast lesions [13] and many drugs whose relative infant dose lies well below the conventional 10% reference point [11].

### 8.2. Breastfeeding with Defined Monitoring

The second level applies where residual uncertainty or risk can be managed while breastfeeding continues, because a potential adverse effect would be detectable and reversible. Breastfeeding continues unchanged, with defined monitoring.

Two conditions define this level. The effect should be recognizable by a parent or clinician without specialized testing, and it should resolve on withdrawal of the agent. Mild sedation, altered feeding behavior and changed stool pattern may satisfy both, although sedation warrants particular caution in neonates, preterm infants and infants with reduced clearance. An effect that would remain silent until irreversible does not qualify, and management then follows the remaining questions of the decision pathway (Section 8.6).

Limited evidence is common in this setting: 54% of medications listed in a major lactation database had no breastfeeding-related safety data [34]. Defined monitoring may be a more proportionate response to such uncertainty than interruption.

### 8.3. Modified Breastfeeding

Exposure is reduced by modifying how the agent is given or how feeding is organized, while the feeding relationship remains intact.

The pharmacological modifications are described in Section 4.5. Two further modifications are anatomical or temporal. Feeding may be restricted to the unaffected breast where a lesion is unilateral, as with herpes simplex confined to one side [13] or, under WHO guidance, mpox lesions affecting one areola [74]. Feeding at the breast may also be suspended while expressed milk is given, where transmission is by a respiratory or contact route and pathogen-specific guidance permits, as in maternal tuberculosis and perinatal varicella [13].

Separating a mother from her infant is not in itself a reason to withhold her milk, unless pathogen-specific guidance requires it.

Cytomegalovirus shows that modification can also apply to the milk itself. The virus is commonly excreted in the milk of seropositive women, beginning in the first postpartum week and peaking at four to eight weeks [100]. Postnatal disease is uncommon in term infants. Infants born before 32 weeks of gestation or weighing less than 1500 g may be at higher risk of symptomatic infection, including a sepsis-like syndrome [100]. In a meta-analysis of such infants born to seropositive mothers, 19% (95% confidence interval, 11–32%) of those fed untreated milk acquired infection and 4% (2–7%) developed the sepsis-like syndrome [100]. With frozen milk, the corresponding figures were 13% (7–24%) and 5% (2–12%) [100]. Freezing lowers viral titers but does not reliably eliminate the virus, and practice varies between neonatal units. The benefits of human milk for these infants appear to outweigh the risk [100]. The decision therefore concerns how the milk is handled and for how long, and the need for treatment may be confined to the period of highest risk [100].

### 8.4. Temporary Interruption

This level may be considered when three conditions are met: exposure cannot be sufficiently reduced (Level 3); the residual risk cannot be acceptably managed by defined monitoring (Level 2); and the applicable agent-, procedure-, radionuclide- or pathogen-specific guidance supports a temporary restriction with an established criterion for resumption. Where guidance supports temporarily withholding both breastfeeding and expressed milk but states no criterion for resumption, as for untreated brucellosis (Section 7.6), the third condition is not met and the situation is not assigned to a level: the restriction is maintained until individualized specialist assessment establishes that breastfeeding can resume, and the criterion applied and its rationale are documented. Three specifications should accompany the decision when temporary interruption is advised.

The first is the indication, stated in terms of the agent and the route. The second is the minimum necessary duration, derived from elimination kinetics, decay or documented resolution of infectivity rather than from convention. The third is the criterion by which resumption will be judged; elimination or decay alone does not establish that breastfeeding may resume (Section 8.5), and the criterion must be one that the applicable guidance supports. Without all three, interruption may function as unintended weaning (Section 3.4).

Maintenance of milk removal throughout the temporary interruption is integral to this level (Section 3.1 and Section 9).

### 8.5. Avoidance or Definitive Cessation

This level applies where exposure is not sufficiently reducible and the applicable guidance recommends avoidance or cessation, without resumption in the relevant clinical circumstances or for the current breastfeeding episode. Membership is small and situation-specific and should be verified against current authoritative resources at the time of the decision [12], since classification changes as evidence accrues. The framework organizes existing guidance here and does not itself create a contraindication.

Three situations illustrate the reasoning. Therapeutic radioiodine concentrates in lactating breast tissue [65]; current guidance regards its administration during ongoing lactation as contraindicated, advises that breastfeeding be stopped before treatment and does not support resumption for the current infant, although the radionuclide eventually decays [7,12,67]. Cytotoxic therapy is regarded as incompatible with breastfeeding during treatment, although agent-specific modeling is beginning to refine that position [12,33,48]. Where maternal HIV infection is not accompanied by sustained viral suppression on antiretroviral therapy, replacement feeding remains the recommendation [13,69].

HTLV-1 shows that the level applied depends on the guidance in force (Section 7.2). Because transmission risk increases with cumulative breastfeeding duration [70,80], the relevant measure, where breastfeeding is permitted at all, is a limit on its duration. Several authorities, CDC among them, advise avoidance of breastfeeding and of expressed maternal milk [13], which corresponds to this level. Others confine duration-limited breastfeeding to settings where replacement feeding is not acceptable, feasible, affordable, sustainable and safe [84].

### 8.6. A Proposed Decision Pathway

The levels can be approached through a conceptual decision pathway. Its first four questions are clinical judgments informed by the determinants in Section 4; the fifth arises only when restriction is required and is answered by the applicable guidance rather than by any single determinant.

The first question asks whether a plausible route of infant exposure exists; if not, breastfeeding can continue without restriction (Level 1). The second asks whether the resulting exposure is clinically meaningful for this particular infant, considering its magnitude and duration, the infant’s gestational and postnatal age, prematurity, organ function and feeding pattern, other susceptibility factors, and the severity and reversibility of potential harm. If it is not, Level 1 applies, or Level 2 where residual uncertainty justifies defined monitoring. The third asks whether exposure can be reduced sufficiently while breastfeeding continues; if so, modified breastfeeding applies (Level 3).

**Figure 8:**
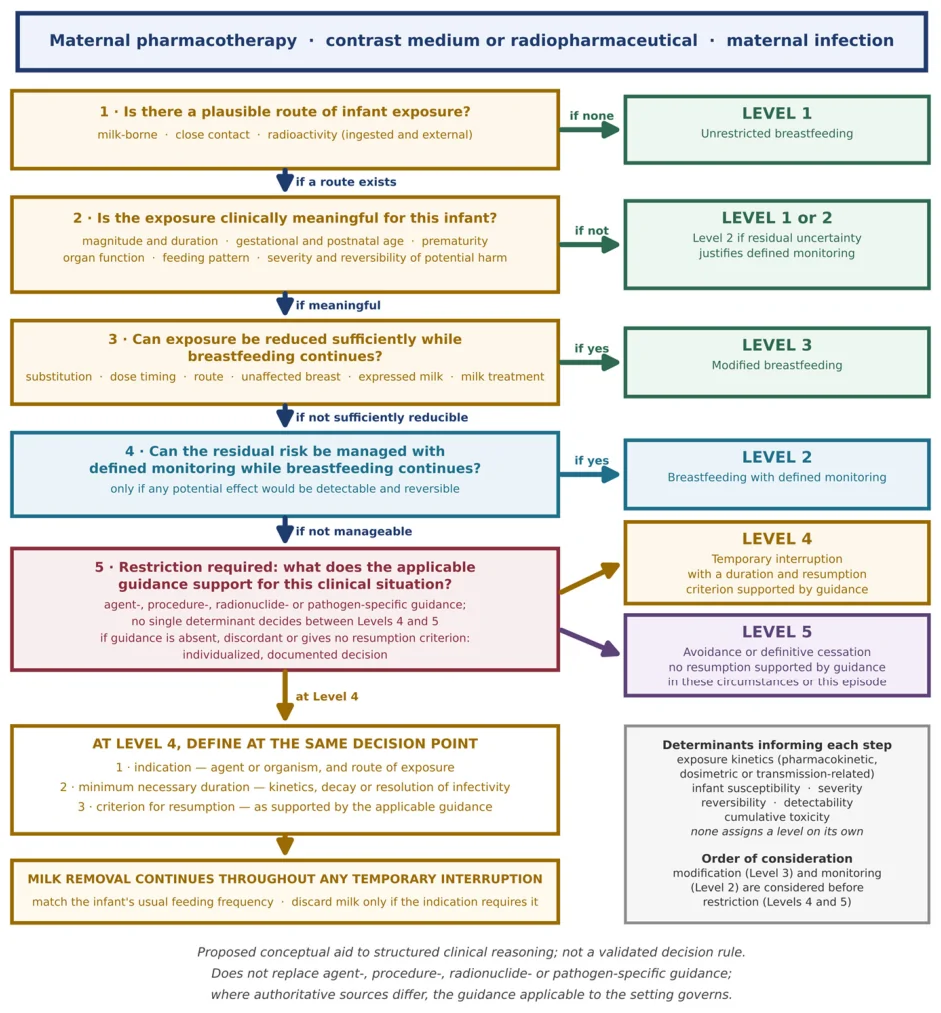
Proposed conceptual decision pathway. The first four questions are clinical judgments informed by the determinants in Section 4, and modification (Level 3) and defined monitoring (Level 2) are considered before restriction. Whether a necessary restriction is temporary (Level 4) or takes the form of avoidance or definitive cessation (Level 5) follows the applicable agent-, procedure-, radionuclide- or pathogen-specific guidance; no single determinant assigns a level. Where guidance is absent or discordant within the setting, or supports temporary withholding of breastfeeding and expressed milk without a criterion for resumption, the decision is individualized and documented (Section 8.4). At Level 4, the indication, minimum necessary duration and criterion for resumption are defined together. Levels are numbered by increasing restriction of the feeding relationship. The pathway is not a validated decision rule and does not replace that guidance.

The fourth question asks whether the residual risk can be acceptably managed while breastfeeding continues with defined monitoring, because any potential effect would be detectable and reversible; if so, Level 2 applies. If restriction is required, the fifth question asks what the applicable agent-, procedure-, radionuclide- or pathogen-specific guidance supports for the clinical situation. Where it supports a temporary restriction with an established criterion for resumption, Level 4 applies, and the indication, the minimum necessary duration and the criterion for resumption are specified at the same decision point. Where it recommends avoidance or cessation, without resumption in the relevant circumstances or for the current breastfeeding episode, Level 5 applies. Severity, reversibility, cumulative toxicity, exposure kinetics and infant susceptibility inform each question, but none assigns a level on its own. Where guidance is absent or discordant within the setting, including where it supports temporarily withholding both breastfeeding and expressed milk but states no criterion for resumption (Section 8.4), the decision is individualized and its basis documented (Figure 8).

Five situations illustrate how the pathway applies, assuming a healthy term infant. They are illustrative applications of current authoritative guidance, not recommendations generated by the framework, and they do not constitute evidence of its validity. After contrast-enhanced computed tomography with an iodinated agent, a route of exposure exists, but the quantity reaching the infant is not clinically meaningful, and breastfeeding continues without restriction (Level 1) [7,50,56]. When a mother requires oral or intravenous clindamycin, current LactMed guidance does not regard this as a reason to discontinue breastfeeding, although an alternative agent may be preferred; if clindamycin is used, breastfeeding continues while the infant is observed for effects on the gastrointestinal flora, such as diarrhea, candidiasis or, rarely, blood in the stool (Level 2) [101]. Herpes simplex lesions confined to one breast are managed by modification: the infant may feed from the unaffected breast, provided that the lesions are completely covered and hand hygiene is observed, whereas feeding from the affected breast and use of its expressed milk are withheld until a clinician confirms that the lesions have healed (Level 3) [13,102]. After a diagnostic administration of technetium-99m pertechnetate, exposure can be neither reduced while breastfeeding continues nor managed by monitoring, and current guidance supports a temporary interruption whose length depends on the source, the administered activity and the procedure: Academy of Breastfeeding Medicine guidance makes the interval depend on the administered activity, with 12 to 24 h, and as little as 4 h after 185 MBq [7], whereas the 2026 American Thyroid Association guideline recommends 36 h, with milk expressed and discarded, after diagnostic thyroid scintigraphy [67]. The cited biokinetic data, by contrast, yield an interval of 12 h derived from dosimetric estimates [64]. Whichever applicable guidance is followed, the indication, the interval and its elapse as the criterion for resumption are specified together (Level 4). Where therapeutic iodine-131 is considered, current guidance regards its administration during ongoing lactation as contraindicated [12,67]. When treatment is required, breastfeeding of the current infant is stopped at least four weeks [7] or 6 to 12 weeks [12] before it, with the American Thyroid Association guideline discussing a wait of at least three months after cessation of lactation [67], and is not resumed despite eventual decay [7,12,67] (Level 5).

The distinction between Levels 4 and 5 was also checked against situations in which the level depends on the clinical circumstances. Cytotoxic therapy corresponds to Level 5 for the duration of treatment [12,48]; a calculated post-dose interval does not by itself move the decision to Level 4 unless the applicable guidance supports it as a criterion for resumption (Section 5.5). For HIV, the level follows the maternal virological state and the guidance in force: where breastfeeding is supported with sustained viral suppression, exposure is reduced at source by antiretroviral therapy and maternal viral load is monitored, which corresponds most closely to Level 3; a detectable viral load may prompt temporary stopping while the cause is assessed (Level 4, where guidance states a criterion for resumption) or discontinuation; and without sustained suppression, replacement feeding is recommended (Level 5) [13,69]. For HTLV-1, avoidance applies where guidance requires it (Level 5) [13], whereas any time limit on breastfeeding elsewhere is set by the guidance applicable in that setting rather than derived from the framework [84]. Tuberculosis and perinatal varicella, in which direct breastfeeding is suspended while expressed milk may be given, remain at Level 3 (Section 8.3). Herpes simplex lesions on both breasts and hepatitis B or C with cracked and bleeding nipples call for guidance-supported temporary restriction with stated criteria for resumption (Level 4) [13,98,102]. Untreated brucellosis is not assigned to a level: the guidance supports temporarily withholding both breastfeeding and expressed milk but states no criterion for resumption [13], so resumption is decided by individualized specialist assessment (Section 8.4). Where guidance differs, so does the level: confirmed Ebola virus disease corresponds to Level 4 under WHO guidance, which states a criterion for resumption [73], and more closely to Level 5 under CDC guidance, which advises against breastfeeding and expressed milk and states no criterion for resumption [13]; mpox corresponds to Level 4 under CDC guidance and to Level 3 under WHO guidance [13,74].

## 9. When Interruption Is Necessary: Duration and Resumption

Once interruption has been judged necessary, its minimum necessary duration and the criterion for resumption should be defined with the indication at the same clinical decision point. Existing guidance addresses these elements inconsistently; the subsections below organize it and do not constitute a new guideline or a validated protocol.

### 9.1. Defining the Indication

A written indication allows an interruption to be reviewed. It should name the agent or organism, the route by which it would reach the infant, and the reason that no measure in Section 4.5 can close that route. Other clinicians can then reassess the decision rather than simply inherit it.

The note to Table 4 describes earlier advice on iodine-123 that may have persisted after its technical basis was questioned. The same can occur for an individual patient, on a shorter timescale.

### 9.2. Defining the Minimum Necessary Duration

Duration should be derived from the indication rather than set by convention, and each domain yields a different quantity.

For drugs, the interval follows maternal elimination kinetics. Where an agent has a short half-life and no accumulation, plasma concentration falls predictably and the interval is calculable [11]. Where the half-life is long or metabolites are active, no feasible interval achieves meaningful reduction; a temporary interruption is then unlikely to help, and management follows agent-specific guidance, which may support monitoring, modification or avoidance.

For radiopharmaceuticals, the interval follows physical decay and biological elimination and is agent-specific. Published dosimetry gives the interval for each compound against an effective-dose criterion of 1 mSv [62,64], whereas society guidance may specify a different interval for a particular procedure, as for diagnostic thyroid scintigraphy (Section 6.4). Expression of milk should not be used to shorten the interval without an agent-specific assessment (Section 6.5).

For infection, the interval follows documented resolution of infectivity rather than the passage of time. In maternal tuberculosis, for example, the criterion is a minimum of two weeks of appropriate treatment together with documentation that the mother is no longer contagious, not two weeks alone [13].

### 9.3. Maintaining Lactation During Interruption

Milk removal should continue throughout any temporary interruption, for the physiological reasons set out in Section 3.1. Frequent and effective removal limits accumulation of the autocrine inhibitor within the gland [18].

The quantitative guidance available comes from a related but distinct population. A state-of-the-science review of mothers dependent on expression for infants in neonatal intensive care assessed 46 eligible studies and drew evidence from 26. It concluded that expressed milk volume may be influenced by several potentially modifiable factors, frequency among them [103]. A threshold of at least eight expressions in 24 h is recommended in that setting, because any break in effective removal may seriously compromise subsequent production [104]. Observational data are consistent: mothers of very low birthweight infants achieving adequate volumes express more than six times daily [104], and in a prospective series of 30 mothers of preterm infants, pumping six or more times daily was associated with reaching full lactation [105]. For comparison, exclusively breastfed infants aged one to six months feed about 11 times daily [30].

These figures concern establishing lactation after preterm delivery, not maintaining an established supply through a brief interruption, and the two are not physiologically equivalent. The direction of the relationship is nonetheless consistent with the mechanism, since the same feedback inhibition operates in both settings [16,17,18]. A reasonable working target is therefore the infant’s usual feeding frequency rather than a fixed number.

### 9.4. Alternative Infant Feeding During Interruption

Whether expressed milk is discarded depends on the indication. Where the concern is a radionuclide, milk expressed during the interval carries activity and is discarded, or in some cases stored until decay renders it usable [7]. Where the concern is maternal contagiousness rather than milk contamination, expressed milk can usually be given, as CDC guidance specifies for tuberculosis and perinatal varicella [13].

Three options exist for feeding during the interval: milk expressed and frozen before a planned exposure, donor human milk where a bank is accessible, and infant formula. The hierarchy is not absolute, and the practical constraint is usually the notice available rather than preference.

### 9.5. Criteria for Resumption

The criterion for resumption should be stated at the same time as the indication, and it differs by domain.

For a drug with sufficiently predictable elimination kinetics, resumption may follow elapse of the agent-specific interval without further testing, where the applicable guidance supports this. For agents with prolonged elimination, clinically relevant active metabolites or substantial accumulation, a calendar-based interval may be inappropriate; management should then follow agent-specific exposure, toxicity and current authoritative guidance. For a diagnostic radionuclide, resumption follows elapse of the interval specified by the applicable guidance for the agent, the administered activity and the procedure, whether derived from dosimetry or set by a professional society, and where sources differ, the guidance applicable to the setting governs [7,62,67]; after therapeutic iodine-131, breastfeeding of the current infant is not resumed [7,12,67]. For maternal infection, resumption generally follows clinical verification rather than a fixed duration, such as documented non-infectiousness after at least two weeks of appropriate treatment in tuberculosis [13] or healing of breast lesions in herpes simplex [13]. Table 6 lists representative criteria by type of indication, including brucellosis, perinatal varicella and mpox.

The distinction matters because resumption after a drug or a diagnostic radionuclide usually follows a calculated or guidance-specified interval, whereas resumption after maternal infection requires clinical verification or guideline-specific reassessment. Advising a woman with breast lesions to resume after a fixed number of days substitutes a duration for a criterion and may be inappropriate in either direction.

### 9.6. Supporting Successful Return to Breastfeeding

Resumption is not assured, and outcomes may depend on the support provided during the interval (Section 3.4) [20,21].

A published case illustrates the support involved. A mother with severe herpetic breast lesions, given expression equipment from the outset, maintained her supply through prolonged healing, resumed breastfeeding and continued to two years [88]. The authors conclude that supporting lactation, not advising cessation, is the appropriate institutional response [88].

A temporary interruption should therefore be accompanied, when advised, by expression equipment and instruction, a named criterion for resumption and scheduled review. Without them, it may be more likely to end in cessation.

## 10. Discussion

### 10.1. Principal Synthesis

In 1990, two clinical pharmacologists observed that transfer into milk had not been studied for many drugs, so mothers were advised against breastfeeding. They added that this advice was in many cases undoubtedly unnecessary, because the dose reaching the infant was often negligible [36]. In 2007, the same observation was made about radiological procedures [106]. By 2019, the evidence gap had been quantified [34], and more recent work has begun to quantify its consequences for breastfeeding [8,9].

Across the three domains, recommendations frequently rest on limited evidence whose limitations are not always stated. Product labeling tends to be more restrictive than lactation-specific resources, which may reflect the scarcity of human lactation data; in one analysis, such data appeared in the labels of 2.8% of recently approved medications [45]. Imaging guidance is permissive, yet its central quantitative assumption, that under 1% of ingested contrast is absorbed, rests on extrapolation from outside the breastfed-infant setting rather than on direct measurement in breastfed infants [53]. Infection guidance can diverge: nineteen European guidelines drawn from the same research reach different conclusions about the same clinical situation [77], and CDC and WHO differ on mpox [13,74].

The direction of the recommendations differs between domains, but the practical difficulty is similar: when sources cannot readily be reconciled, interruption may appear to be the cautious default. The proposed framework seeks to make that choice explicit, so that interruption, when advised, rests on a stated indication.

Table 7 summarizes the evidential basis of the principal propositions of this review. It distinguishes recommendations reproduced from authoritative guidance from propositions synthesized by the authors and states the principal limitation of each. Direct infant-outcome evidence contributes mainly to the propositions on maternal infection. Most propositions rest on pharmacokinetic or dosimetric measurement, transmission data, authoritative guidance or physiological reasoning, and the five-level framework itself is reasoning-based.

### 10.2. Positioning Against Existing Literature and Guidance

Previous work established the components of this argument separately. The physiology of autocrine control has been characterized since the 1990s [16,17,18]. The inadequacy of the milk-to-plasma ratio in isolation, and the dependence of infant exposure on clearance, were set out in 1994 [29]. A 2025 systematic review suggested that much medication-related discontinuation may be avoidable [9], and surveys have documented practice variation after imaging [10] and after the revision of perinatal HIV guidance [79].

This review brings these studies into a single decision structure. It addresses how the decision itself is made, applies the same sequence of questions to contrast media, radiopharmaceuticals and maternal infection, and makes resumption part of the initial decision. Milk4baby, developed within the ConcePTION project, has a different aim: it guides the choice of methods for studies of infant exposure to medications through human milk [14]. The present framework addresses clinical decisions while such evidence remains incomplete.

The relationship of the framework to current recommendations follows from this difference in purpose. Current guidance and lactation resources are, by design, specific to an agent, a disease, a procedure or a radionuclide: LactMed and product information address individual medicines [12], radiology and nuclear medicine guidance addresses contrast media and radiopharmaceuticals [7,50,56,62,63], and public health guidance addresses individual pathogens [13,69,73,74]. Some resources already group recommendations into categories. CDC guidance, for example, distinguishes conditions in which breastfeeding and expressed milk are avoided, conditions in which both are temporarily withheld, and conditions in which direct breastfeeding is suspended while expressed milk may still be given [13]. WHO guidance from 2009 grouped maternal conditions, including infections and medicines such as radioactive iodine-131, by whether breastfeeding could continue or should be avoided temporarily or permanently [107]. It is cited here as a historical precedent: its advice that breastfeeding may resume about two months after iodine-131 differs from current guidance on iodine-131 sodium iodide, which, under the conditions it specifies, advises that breastfeeding not be resumed for the current infant [7,12,67]. Other resources grade individual agents: Hale’s lactation risk categories rate medicines [108], and e-lactancia rates medicines, procedures and maternal conditions on a four-level compatibility scale [109]. These resources classify agents and conditions. The present framework does not replace them; it describes clinical actions for a particular mother-infant dyad and provides a cross-domain structure for clinical reasoning that links three elements of one decision: whether interruption is necessary, the minimum necessary duration when restriction is required, and the criterion for resumption. Among the sources identified for this review, we did not find another framework linking these elements across the three domains, although the search was not designed to establish that none exists (Section 10.4). The framework assigns no management level independently of current guidance, which remains determinative for the individual clinical situation; where authoritative sources differ, the guidance applicable to the setting governs.

### 10.3. Evidence Gaps and Research Priorities

Several evidence gaps apply across the domains. Lactating women remain largely excluded from clinical trials, so most agents reach the market without human lactation data [45]. Infant pharmacokinetic and pharmacodynamic data are scarcer still, and relative infant dose compares infant exposure with the maternal rather than the neonatal therapeutic dose, which is usually unknown. Developmental changes in drug disposition remain little studied, particularly in preterm and medically fragile infants, in whom reduced clearance matters most [11,31].

Three gaps are domain-specific. Gastrointestinal absorption of contrast media from milk has not been measured directly in breastfed infants, although the extrapolated figure is widely quoted as established [53]. Radiopharmaceutical activity concentrations in milk span seven orders of magnitude [64], which makes agent-level dosimetry necessary. The intersection of liberalized HIV guidance with unrecognized HTLV-1 carriage is an emerging risk that antenatal screening rarely addresses [85].

Two further priorities follow from this review directly. Criteria for interruption and for resumption are not standardized across the domains examined, and the framework proposed in Section 8 has not been evaluated in clinical practice.

Such evaluation could proceed in two stages. The first would examine reproducibility, feasibility and usability. Clinicians from different specialties would apply the framework to standardized scenarios spanning the three domains. Agreement between clinicians in the management level, an ordinal category, could be quantified with weighted kappa; agreement in the criterion for resumption, a nominal category, with Cohen’s or Fleiss’ kappa; and agreement in the proposed duration of interruption, a continuous measure, with the intraclass correlation coefficient or Bland–Altman limits of agreement. Agreement with an independent expert panel could also be assessed, the panel’s judgment serving as a reference assessment rather than as evidence of clinical benefit, together with the completeness of documentation, the time needed to reach a decision and clinicians’ ratings of usability. This stage would show whether the framework can be applied consistently, not whether its use improves outcomes.

The second stage would examine the effects of using the framework in practice, for example in a prospective multicenter before-and-after or stepped-wedge implementation study. Candidate outcomes would include the appropriateness of interruption, judged by reviewers unaware of the study period, rather than its frequency alone, because the framework is intended both to avoid unnecessary interruption and to support restriction where it is indicated; the duration of interruption relative to the indication; continuation or successful resumption of breastfeeding at prespecified time points; unintended permanent cessation; maternal lactation complications; and maternal treatment or imaging delayed or declined because of breastfeeding. Concordance with authoritative guidance would be a necessary reference point but not an absolute standard, because guidance differs between authorities for several of the situations examined here. Infant adverse outcomes are essential safety outcomes; because serious events are rare, their assessment would require linkage to sufficiently large, appropriately designed prospective surveillance rather than reliance on implementation studies of conventional size.

### 10.4. Limitations

Several limitations qualify these conclusions. The review is not systematic: the clinical domains were defined in advance, but selection within them was purposive and reflects the authors’ judgment rather than prespecified eligibility criteria, and the infections in Section 7 were chosen to illustrate distinct decisional principles, not to be exhaustive. For the reason given in Section 2.4, recommendations carry no formal certainty rating. The framework in Section 8 has not been prospectively, externally or outcome-validated, nor subjected to formal consensus. Quantitative guidance on expression frequency derives from mothers establishing lactation after preterm delivery or the admission of their infants to neonatal intensive care, not maintaining it through a planned temporary interruption [103,104]. The relactation figures likewise derive mainly from mothers of hospitalized or non-breastfed infants, in whom reasons for stopping were rarely reported [21].

The language restriction and the purposive selection have further consequences. Sources were limited to English, French and Romanian, and all sources ultimately cited were published in English; guidance and data available only in other languages, including national recommendations, may therefore be under-represented. Database-specific search strings and record counts are not reported, so the search cannot be reproduced exactly. Because it was not designed to establish that no comparable framework exists, the absence of one among the sources identified does not exclude its existence, particularly in languages not searched. Because a narrative review depends on the authors’ judgment of relevance, pertinent studies may have been missed, and the focus of this review on unnecessary interruption may have given more weight to evidence of over-restriction than to evidence of under-restriction. The evidence that interruption or discontinuation is advised more often than necessary comes from a limited number of settings [8,9,10,57], and much of the infection guidance examined was issued in the United States [13,69]. Guidance in several of the areas reviewed, including HIV and mpox, is revised periodically, so the recommendations cited here should be checked against the version current at the time of a decision.

The generalizability of the framework should be read in the same light. The reasoning structure was designed to be applicable across clinical settings, although this transferability has not yet been empirically evaluated; individual management decisions remain context-dependent. They depend on the infant’s gestational and postnatal age, prematurity, organ function, concurrent illness and the extent of breastfeeding (Section 4.3 and Section 5.6); on local epidemiology and the national or international guidance that applies; and on resources that differ between health systems, including laboratory monitoring, lactation support, donor human milk, safe replacement feeding and expertise in nuclear medicine dosimetry. Where safe replacement feeding cannot be assured, interruption carries greater risk for the infant, and the balance between options shifts accordingly [3]. A decision that is appropriate in one setting cannot therefore be assumed to apply unchanged in another.

A further limitation concerns the scope of the argument. This review focuses on settings in which interruption or discontinuation may be advised more readily than the evidence warrants, as documented for medication use and diagnostic imaging [8,9,10]. Interruption may also be under-applied, as when more permissive HIV guidance meets unrecognized HTLV-1 infection (Section 7.2) [85]. The two risks have been summarized elsewhere: if breastfeeding is wrongly discouraged, years of public health effort may be lost; if wrongly encouraged, infants may be put at risk [96]. Both errors carry real costs, and the focus of this review on the former should not be interpreted as minimizing the clinical consequences of inappropriate continuation.

## 11. Conclusions

Three quantities are often conflated in this field: the intrinsic capacity of an agent to cause harm, the fraction of it that reaches the infant, and the probability that this fraction produces a detectable effect. Recommendations based predominantly on intrinsic hazard may overestimate the need for breastfeeding interruption. Observational studies and clinician surveys indicate that unnecessary interruption or discontinuation of breastfeeding occurs in several clinical settings, particularly medication use and diagnostic imaging [8,9,10].

Interrupting breastfeeding is a clinical intervention in its own right. Without maintained milk removal, it may downregulate milk synthesis and increase the risk of unintended permanent cessation, particularly where structured resumption support is not provided. Across maternal pharmacotherapy, contrast media and radiopharmaceuticals, and selected maternal infections, the same questions lead from maternal exposure to infant clinical risk, although each domain answers them with its own quantities. Applied consistently, these questions may help to identify both the situations that can be managed without interruption and those in which restriction is warranted.

When temporary interruption is necessary, the indication, the minimum necessary duration and a criterion for resumption supported by the applicable guidance should be defined at the same decision point, and milk removal should continue throughout. The proposed framework organizes existing guidance, does not replace agent-, procedure-, radionuclide- or pathogen-specific recommendations, and requires prospective evaluation of its reproducibility and of its effects on breastfeeding and infant outcomes.

## Figures and Tables

**Table 1:** Conceptual physiological and behavioral consequences of breastfeeding interruption across illustrative duration bands.

Illustrative Duration Band	Effect on Milk Synthesis	Stasis and Inflammation	Feeding Behavior	Resumption
Under 24 h, removal maintained	Suppression may remain limited if the feedback inhibitor is cleared at each expression	Risk may remain comparatively low while effective milk removal continues	Feeding behavior may remain unaffected if direct breastfeeding resumes promptly	Conditions may favor resumption
Under 24 h, removal not maintained	Alveolar retention may begin to suppress synthesis	Stasis and inflammatory complications may become more likely if removal is inadequate	Breast refusal may occur after bottle introduction	Resumption remains plausible; additional lactation support may be needed
24–72 h	Risk of reduced supply may increase; in direct measurement, intervals of 6–18 h yielded proportionally less milk than intervals under 6 h	Risk across the mastitis spectrum may increase	Bottle preference may become more relevant	Structured lactation support may be required
Over 72 h	Sustained downregulation may become increasingly likely	Inflammatory risk may increase further if removal is inadequate	Breast refusal may become increasingly relevant as the gap lengthens	Probability of direct resumption may decline as the gap lengthens
Definitive	Involution	Resolves with involution	Not applicable	Relactation would be required, with outcome dependent on support, infant age and length of the gap

Framework note. The duration bands are an illustrative structure proposed by the authors and have not been validated as clinical thresholds. The physiological relationships within each band are sourced, whereas the band boundaries are not taken from any single study. Entries give the expected direction of effect and do not quantify probability. Sources: autocrine control and the effect of removal interval [16,17,18]; mastitis spectrum [19]; bottle feeding and breast refusal [10]; relactation 39.4% in a general population survey [20] against over 80% in 13 of 16 studies with structured support [21].

**Table 2:** The evidential basis of lactation guidance across source types.

Source Type	Evidential Basis	Documented Finding	Potential Interpretive Tendency
Lactation databases	Published human lactation studies where available	Of 1408 medications listed, 54% had no breastfeeding safety data and 2% had sound evidence [34]	States the absence of data explicitly
Product labeling	Regulatory submission data, frequently animal only	Of 290 medications approved 2010–2019, 2.8% of labels contained human lactation data and 10.7% human pregnancy data; 45 of 138 (32.6%) products submitted before the 2015 labeling rule took effect were not in the required format by June 2019 [45]	May favor conservative recommendations when direct human lactation data are unavailable
Comparison across resources	Differing interpretation of limited human evidence	Recommendations for 19 medications across 10 drug information resources varied by source; dedicated lactation references classified the most agents as compatible [44]	Divergent; may reflect different evidentiary thresholds and update cycles
Animal milk statements on labels	Presence or absence of drug in animal milk	Judged largely irrelevant to human lactation safety, since risk is governed by quantity rather than by detection [28]	Conservative wording not matched by human evidence

**Table 3:** Proposed graded management levels, defined by clinical action rather than by pharmacological class.

Level	Defining Criterion	Feeding	Additional Measure	Documentation Suggested
Unrestricted breastfeeding	No plausible exposure route, or quantified exposure very low with no qualifying consideration from Section 4.4	Unchanged	None	Document the absence of clinically meaningful exposure where relevant
Breastfeeding with defined monitoring	Residual uncertainty or risk manageable during continued breastfeeding; potential effect detectable and reversible	Unchanged	Defined monitoring for sedation, altered feeding or altered stool pattern	Specify the adverse effects to be monitored
Modified breastfeeding	Exposure reducible at source or by feeding organization	Continues	Substitution, dose timing, route change, unaffected breast only, expressed milk during separation, milk treatment in selected preterm infants	Document the exposure-reduction strategy adopted
Temporary interruption	Exposure not sufficiently reducible; residual risk not manageable by monitoring; applicable guidance supports temporary interruption of justified duration with an established resumption criterion	Suspended until the stated resumption criterion is met	Milk removal maintained; stated resumption criterion	Document indication, minimum necessary duration, milk-removal plan and resumption criterion
Avoidance or definitive cessation	Exposure not sufficiently reducible; residual risk not manageable by monitoring; applicable guidance recommends avoidance or cessation and does not support resumption in the relevant circumstances or for the current breastfeeding episode	Avoided or definitively ceased according to applicable guidance	Alternative feeding established	Verify against current agent-, procedure-, radionuclide- or pathogen-specific guidance and document the rationale for avoidance or cessation

Note. This is a proposed conceptual organization of existing guidance, not a validated clinical decision rule. Severity, reversibility, cumulative toxicity, exposure kinetics and infant susceptibility inform the assessment at every level, but none assigns a level on its own; whether a necessary restriction is temporary (Level 4) or takes the form of avoidance or definitive cessation (Level 5) follows the applicable guidance. No agent names appear in the table, because classification changes as evidence accrues and agent-level determinations should be made against a current authoritative lactation resource such as LactMed [12] rather than from a list published in a review. Some pathogen-specific guidance permits duration-limited breastfeeding in defined settings; such cases remain guidance-dependent and are not defined by the framework (Section 8.5). Where guidance supports temporarily withholding both breastfeeding and expressed milk but states no criterion for resumption, the situation is not assigned to a level, and resumption is decided by individualized specialist assessment (Section 8.4).

**Table 4:** Infant exposure and recommendations on interruption, presented in two panels because the governing quantities differ: (**a**) contrast media—governed by the quantity transferred and absorbed, for which the decision turns on the quantity transferred into milk and absorbed from the infant gut; (**b**) radiopharmaceuticals—governed by administered activity, biokinetics and dosimetry, for which it turns on administered activity, biokinetics and radiation dosimetry.

(a)				
Agent Class	Excreted into Milk, 24 h (Measured)	Absorbed from the Infant Gut (Assumed)	Estimated Infant Systemic Exposure (Calculated)	Interruption (Guidance)
Iodinated contrast media	Under 1% of the maternal intravascular dose [50]	Under 1% of the ingested quantity; extrapolated, not measured in breastfed infants [50,53]	Under 0.01% of the maternal intravascular dose [50]	None [50]
Gadolinium-based agents	Under 0.04% of the maternal intravascular dose, measured with gadopentetate dimeglumine, a linear agent [50,58]	Under 1% of the ingested quantity; same extrapolation [50,53]	Under 0.0004% of the maternal intravascular dose [50]	None after macrocyclic agents [56]
(**b**)				
**Agent or Group**	**Activity Excreted into Milk (Measured)**	**Determinants of Infant Exposure**	**Estimated Infant Effective Dose (Dosimetric Model)**	**Interruption and Contact Precautions (Source Recommendations)**
Technetium-99m compounds	0.0057% of administered activity for labeled red cells to 19% for pertechnetate [64]	Physical decay and biological elimination; ingestion of excreted activity [62], plus external dose from retained maternal activity at close range [65,66]	6.7 × 10^−6^ to 3.6 × 10^−2^ mSv per MBq administered to the mother [64]	Derived from dosimetric estimates in the cited biokinetic dataset: none for most compounds and 12 h for pertechnetate and for macroaggregated albumin, with milk expressed and discarded during the interval [64]; current guidance for pertechnetate: 12 to 24 h depending on administered activity, and as little as 4 h after 185 MBq [7]; 36 h, with milk expressed and discarded, after diagnostic thyroid scintigraphy [67]
Other diagnostic radiopharmaceuticals	0.018% of administered activity for chromium-51 ethylenediaminetetraacetic acid upward; activity per milliliter of milk spans 10^−10^ to 10^−3^ MBq/MBq across all 16 agents studied [64]	As above, and determined agent by agent rather than by imaging modality [62,63,64]	5.6 × 10^−5^ mSv per MBq administered upward [64]	Derived agent by agent from published dosimetry against a 1 mSv effective-dose criterion [62,63,64]; interruption judged necessary for 9 of 25 agents in an independent review [65]
Iodine-131 sodium iodide	Up to 48% of administered activity [64]	Concentrates in lactating breast tissue; prolonged physical and biological retention	Up to 106 mSv per MBq administered [64]	Cessation [64]; current guidance: contraindicated during lactation [12,67]; breastfeeding stopped at least 4 weeks [7] or 6 to 12 weeks [12] before therapy to reduce the radiation dose to the breast, and a wait of at least 3 months after cessation of lactation discussed in thyroid guidance [67]; not resumed for the current infant [7,12,67]

Evidential caveat. No direct measurement of gastrointestinal absorption of contrast media in breastfed infants was identified; the widely cited figure of under 1% is therefore an extrapolated assumption, and a PubMed keyword search located only reviews and guidelines mentioning it in their discussion [53]. Measured iodine concentrations in milk span 0 to 36.5 mg/L over 24 h [55]. Published milk measurements derive from 22 women given iodinated agents, most of them agents no longer marketed, and from women given gadopentetate dimeglumine, a linear gadolinium-based agent; measurements for macrocyclic agents were not identified [53,54]. Note. Entries in panel (**b**) describe the recommendations of the cited biokinetic and dosimetric sources, except where current guidance is specified, and are not presented as universal contemporary guidance; current society recommendations remain tracer-specific and may specify longer intervals than these sources, including for agents that they cover [67,68]. Agent-by-agent intervals for the radiopharmaceuticals requiring interruption are tabulated in Annex D of Publication 106 of the International Commission on Radiological Protection [62] and should be checked against current guidance rather than taken from earlier compilations. For technetium-99m pertechnetate, the panel distinguishes an interval derived from dosimetric estimates in one biokinetic series [64], the activity-dependent recommendation of the Academy of Breastfeeding Medicine [7] and the procedure-specific recommendation of the 2026 American Thyroid Association guideline for diagnostic thyroid scintigraphy [67]; these rest on different assumptions and clinical scope and are not interchangeable. Where cessation is warranted, the two iodine radionuclides must be distinguished. Therapeutic iodine-131 concentrates in lactating breast tissue [65], and current guidance addresses three distinct points: administration during ongoing lactation is contraindicated [12,67]; breastfeeding is stopped at least 4 weeks [7] or 6 to 12 weeks [12] before administration to reduce the radiation dose to the breast, and the American Thyroid Association guideline discusses waiting at least 3 months after cessation of lactation, within an individualized multidisciplinary assessment [67]; and breastfeeding is not resumed for the current infant after treatment [7,12,67]. Diagnostic iodine-123 sodium iodide is a different agent. A 2000 dosimetric review suggested complete cessation on the assumption of 2.5% contamination with iodine-125, a value reported in a single case, whereas without contamination a 24 h interruption sufficed [65]; Publication 106 advised more than 3 weeks because of possible contamination with other iodine isotopes [62]; the Academy of Breastfeeding Medicine notes that recommendations range from no interruption to 3 weeks and that the earlier concern about contamination has likely been resolved [7]; and, for diagnostic thyroid scintigraphy, the American Thyroid Association guideline recommends that milk be expressed and discarded for 3 to 4 days [67].

**Table 5:** Selected maternal infections as decisional archetypes.

Infection	Principle Illustrated	Transmission Route	Feeding at Breast (Guidance)	Expressed Milk (Guidance)	Criterion for Resumption (Guidance)
HIV	Risk modified by treatment	Milk-borne	Supported with consistent ART and sustained viral suppression, including viral load below 50 copies/mL for at least 3 months before delivery [13,69]	Same condition	Not a duration; a maintained state
HTLV-1	Cumulative exposure; management is guideline-dependent	Milk-borne	Guideline-dependent: avoidance of breastfeeding under CDC [13]; exclusive formula feeding preferred elsewhere, with duration-limited breastfeeding an alternative only where replacement feeding is not acceptable, feasible, affordable, sustainable and safe (Section 7.2). Meta-analysis of observational data: pooled relative risk 0.72 (95% CI 0.30–1.77) at ≤3 months and 2.91 (95% CI 1.69–5.03) at ≤6 months, each relative to exclusive formula feeding [70]	Same condition	Set by the applicable current guidance; where breastfeeding is time-limited, adherence to the limit may require active follow-up
Tuberculosis	Contagiousness versus milk safety	Airborne	Suspended while infectious	Permitted throughout; milk is not a recognized transmission route in the absence of breast involvement [13,71]	Minimum two weeks of treatment and documented non-infectiousness [13]
Herpes simplex	Anatomical site determines management	Direct lesion contact	Unaffected breast only	Withheld from affected side until healed [13,72]	Lesion healing
Varicella (perinatal)	Narrow temporal window	Contact, with insufficient transplacental antibody	Suspended if rash 5 days before to 2 days after delivery [13]	Permitted throughout [13]	Not specified in the guidance [13]; clinical judgment that the mother is no longer infectious
Brucellosis (untreated)	Temporary restriction without a stated resumption criterion	Milk-borne	Suspended	Suspended	Not specified in the guidance [13]; initiation of treatment alone does not establish that breastfeeding can resume; individualized specialist assessment
Ebola virus disease	Documented outcome; stopping, with a resumption criterion under WHO guidance	Milk-borne and other fluids	WHO: stopped where infection is suspected or confirmed [73]; CDC: avoided [13]	Stopped	WHO: two consecutive breast milk RT-PCR tests negative 24 h apart [73]; CDC: none stated [13]
Mpox	Guideline discordance under limited evidence	Close contact with lesions; role of milk uncertain	CDC: delayed until isolation criteria are met [13]; WHO: conditionally continued with infection-prevention measures, not from a breast with areolar lesions [74]	CDC: withheld [13]; WHO: may be given where safe and feasible, without direct contact [74]	Full resolution of lesions with intact skin [13,74]

Abbreviations: ART, antiretroviral therapy; CDC, Centers for Disease Control and Prevention; CI, confidence interval; HIV, human immunodeficiency virus; HTLV-1, human T-lymphotropic virus type 1; RT-PCR, reverse transcription polymerase chain reaction; WHO, World Health Organization. Note. These infections were selected as decisional archetypes representing distinct transmission pathways and management principles, not as a representative or exhaustive sample of maternal infection. Management in the individual case follows current authoritative guidance for the specific pathogen.

**Table 6:** Criteria for resumption, by type of indication.

Indication	Derivation of Duration	Criterion for Resumption	Basis of the Criterion
Maternal drug, short half-life	Maternal elimination kinetics	Elapse of the agent-specific interval derived from elimination kinetics	Pharmacokinetic calculation, where supported by the applicable guidance
Maternal drug, long half-life or active metabolites	A short interruption may not meaningfully reduce exposure	No fixed interval; management follows agent-specific exposure and toxicity data and applicable guidance	Agent-specific guidance (monitoring, modification or avoidance, depending on the agent)
Radiopharmaceutical	Physical decay and biological elimination; in dosimetric sources, a 1 mSv effective-dose criterion	Elapse of the tracer-, activity- or procedure-specific interval specified by the applicable guidance	Dosimetric calculation or society guidance; not shortened by expression alone
Therapeutic radioiodine	Not applicable (cessation)	No resumption for the current infant	Agent-specific guidance [7,12,67]
Tuberculosis	Response to appropriate treatment	At least two weeks of appropriate treatment and physician documentation of non-infectiousness	Clinical verification: documented non-infectiousness
Herpes simplex of the breast	Lesion healing	Healing of lesions on the affected breast	Clinical verification of healing
Brucellosis	Not specified; response to treatment assessed individually	Not specified in the cited guidance; not established by initiation of treatment alone	Individualized specialist assessment, documented
Mpox	Lesion resolution	Full resolution of lesions with intact skin; management before this point differs between CDC and WHO guidance	Clinical verification; guidance-dependent
Perinatal varicella	Maternal infectiousness; the perinatal window defines neonatal risk, not the resumption point	Resolution of maternal infectiousness; timing not specified in the cited guidance	Clinical judgment

Note. The table distinguishes pharmacokinetic or dosimetric intervals from criteria that require clinical verification or guideline-specific reassessment and from situations in which the guidance leaves the timing of resumption to clinical judgment, and it organizes existing guidance and the sources on which it draws [7,12,13,62,64,67,71,74] without itself defining resumption criteria or thresholds. Where authorities differ, as for mpox, the guidance applicable to the setting governs.

**Table 7:** Evidential basis of the principal propositions of the review.

Principal Proposition	Status	Main Evidential Basis	Principal Limitation	Section
Interrupting breastfeeding without maintained milk removal can reduce milk synthesis and compromise resumption	Authors’ synthesis	Human physiological studies of milk synthesis and removal [16,17]; mechanistic animal data [18]; observational data on interruption and relactation [20,21]	Small physiological samples; relactation data from populations other than those considered here	Section 3
Interruption or discontinuation of breastfeeding may be advised more readily than the evidence warrants, particularly after medication use and diagnostic imaging	Authors’ synthesis	Prospective cohort [8], systematic review [9] and clinician surveys [10,57]	Limited number of settings; partly self-reported practice	Section 1, Section 3.2 and Section 5.1
Hazard, infant exposure and clinical risk are distinct; exposure is interpreted with infant susceptibility and the severity and reversibility of potential harm	Authors’ synthesis	Pharmacokinetic principles and human milk-transfer studies [11,28,29]; developmental pharmacology [31]	Reference values, such as a relative infant dose below 10%, are conventions rather than outcome-derived thresholds; data in neonates and preterm infants are scarce	Section 4
Measures that reduce exposure are considered before interruption	Authors’ synthesis	Pharmacokinetic reasoning [27]; pathogen-specific guidance on unilateral lesions and on expressed milk during separation [13]	No comparative studies of exposure reduction versus interruption	Section 4.5 and Section 8.3
Decisions on individual maternal medicines are made against a current lactation resource	Existing guidance	Human pharmacokinetic studies, case reports and pharmacovigilance data compiled in lactation databases [12]	54% of listed medications had no breastfeeding-related safety data; resources may classify the same agent differently [34,44]	Section 5
Breastfeeding continues without interruption after iodinated and gadolinium-based contrast media	Existing guidance	Human milk-transfer measurements with iodinated agents [51,52,55] and gadopentetate dimeglumine [58,59,60]; assumed low oral absorption; guidance [50,56]	Small, largely historical series; infant gastrointestinal absorption not measured; macrocyclic agents not measured in human milk	Section 6.1 and Section 6.2
Interruption after radiopharmaceuticals is agent-specific, with intervals derived from dosimetry against a 1 mSv effective-dose criterion or specified by society guidance, which may be activity- or procedure-specific	Existing guidance	Human biokinetic measurements in milk and radiation dosimetry [62,63,64]; society guidance [7,67,68]	Typical administered activities assumed; considerable inter-individual variation; recommended intervals differ between authorities for some agents [62,64,65,67,68]	Section 6.3, Section 6.4, Section 9.2 and Section 9.5
In maternal infection, management follows the transmission route, and expressed milk may be given during separation where pathogen-specific guidance permits	Existing guidance	Public health guidance [13]; one randomized trial for HIV [75]; cohort and meta-analytic data for HTLV-1, varicella and cytomegalovirus [70,90,100]; case reports and small series for other pathogens	Direct infant-outcome data sparse for several pathogens; guidance differs between authorities, as for mpox and HTLV-1	Section 7 and Section 8.3
When interruption is advised, the indication, the minimum necessary duration and the criterion for resumption are specified together, and milk removal is maintained at about the infant’s usual feeding frequency	Authors’ synthesis	Physiological reasoning [16,17,18]; expression-frequency data from mothers of preterm or critically ill infants [103,104,105]; relactation outcomes with structured support [21]	Not evaluated in clinical studies; expression data extrapolated from establishing lactation after preterm birth	Section 8.4 and Section 9
Five-level framework and decision pathway	Authors’ synthesis	Conceptual integration of the determinants in Section 4 and of the guidance reviewed in Section 5, Section 6 and Section 7	Reasoning-based; not prospectively, externally or outcome-validated	Section 8

Note. Status distinguishes recommendations reproduced from authoritative guidance from propositions synthesized by the authors. Entries describe the main type of evidence and its principal limitation; they are not certainty ratings and do not rank the propositions.

## Data Availability

No new data were created or analyzed in this study. Data sharing is not applicable to this article.

## Abbreviations

The following abbreviations are used in this manuscript:

ART	antiretroviral therapy
BCRP	breast cancer resistance protein
CDC	Centers for Disease Control and Prevention
CI	confidence interval
EDTA	ethylenediaminetetraacetic acid
HHS	U.S. Department of Health and Human Services
HIV	human immunodeficiency virus
HSV	herpes simplex virus
HTLV-1	human T-lymphotropic virus type 1
HTLV-2	human T-lymphotropic virus type 2
IgG	immunoglobulin G
M/P	milk-to-plasma ratio
RT-PCR	reverse transcription polymerase chain reaction
SANRA	Scale for the Assessment of Narrative Review Articles
TB	tuberculosis
VZV	varicella-zoster virus
WHO	World Health Organization
